# Fine mapping of a linkage peak with integration of lipid traits identifies novel coronary artery disease genes on chromosome 5

**DOI:** 10.1186/1471-2156-13-12

**Published:** 2012-02-27

**Authors:** Daniel K Nolan, Beth Sutton, Carol Haynes, Jessica Johnson, Jacqueline Sebek, Elaine Dowdy, David Crosslin, David Crossman, Michael H Sketch, Christopher B Granger, David Seo, Pascal Goldschmidt-Clermont, William E Kraus, Simon G Gregory, Elizabeth R Hauser, Svati H Shah

**Affiliations:** 1 Center for Human Genetics, Duke University, 905 S. LaSalle Street, Duke Univeristy Medical Center, Durham NC, 27710, USA; 2 Department of Medicine, Duke University, 2301 Erwin Road, Durham NC, 27710, USA; 3 Miller School of Medicine, University of Miami, 1601 Northwest 12th Avenue, Miami FL, 33136, USA; 4 University of East Anglia, Norwich Research Park, Norwich, NR4 7TJ, UK

**Keywords:** Cardiovascular Disease, Positional Cloning, Intermediate Phenotype, Linkage, Fine Mapping

## Abstract

**Background:**

Coronary artery disease (CAD), and one of its intermediate risk factors, dyslipidemia, possess a demonstrable genetic component, although the genetic architecture is incompletely defined. We previously reported a linkage peak on chromosome 5q31-33 for early-onset CAD where the strength of evidence for linkage was increased in families with higher mean low density lipoprotein-cholesterol (LDL-C). Therefore, we sought to fine-map the peak using association mapping of LDL-C as an intermediate disease-related trait to further define the etiology of this linkage peak. The study populations consisted of 1908 individuals from the CATHGEN biorepository of patients undergoing cardiac catheterization; 254 families (N = 827 individuals) from the GENECARD familial study of early-onset CAD; and 162 aorta samples harvested from deceased donors. Linkage disequilibrium-tagged SNPs were selected with an average of one SNP per 20 kb for 126.6-160.2 MB (region of highest linkage) and less dense spacing (one SNP per 50 kb) for the flanking regions (117.7-126.6 and 160.2-167.5 MB) and genotyped on all samples using a custom Illumina array. Association analysis of each SNP with LDL-C was performed using multivariable linear regression (CATHGEN) and the quantitative trait transmission disequilibrium test (QTDT; GENECARD). SNPs associated with the intermediate quantitative trait, LDL-C, were then assessed for association with CAD (i.e., a qualitative phenotype) using linkage and association in the presence of linkage (APL; GENECARD) and logistic regression (CATHGEN and aortas).

**Results:**

We identified four genes with SNPs that showed the strongest and most consistent associations with LDL-C and CAD: *EBF1*, *PPP2R2B*, *SPOCK1*, and *PRELID2*. The most significant results for association of SNPs with LDL-C were: *EBF1*, rs6865969, p = 0.01; *PPP2R2B*, rs2125443, p = 0.005; *SPOCK1*, rs17600115, p = 0.003; and *PRELID2*, rs10074645, p = 0.0002). The most significant results for CAD were *EBF1*, rs6865969, p = 0.007; *PPP2R2B*, rs7736604, p = 0.0003; *SPOCK1*, rs17170899, p = 0.004; and *PRELID2*, rs7713855, p = 0.003.

**Conclusion:**

Using an intermediate disease-related quantitative trait of LDL-C we have identified four novel CAD genes, *EBF1*, *PRELID2*, *SPOCK1*, and *PPP2R2B*. These four genes should be further examined in future functional studies as candidate susceptibility loci for cardiovascular disease mediated through LDL-cholesterol pathways.

## Background

Coronary artery disease (CAD) is the end result of accumulation of atheromatous plaques in the coronary arteries, leading to eventual impairment of cardiac blood flow and potentially devastating consequences of myocardial infarction (MI) or death. CAD is the leading cause of death in both the United States and worldwide, with over 500,000 deaths per year in the U.S. and over seven million worldwide (World Health Organization) [1]. Despite the development of pharmacologic therapies for prevention, the incidence of CAD is increasing, concomitant with the rising prevalence of risk factors such as obesity and diabetes (American Heart Association) [2].

CAD itself is clearly a heritable trait, with the role of genetic factors becoming increasingly apparent in early-onset CAD [3-5]. However, the genetic architecture of CAD, as with many common diseases, is assumed to be complex and continues to be poorly understood. Candidate gene studies have identified several loci for CAD, but with inconsistent results in validation cohorts. Recent genome wide association studies (GWAS) have consistently identified a locus on chromosome 9p21; however, this locus confers only modest risk of disease with effect sizes of 1.3-1.6 [6]. Thus, much of the genetic architecture underlying the heritability of CAD remains to be elucidated. There are many well-established risk factors for CAD that are partitioned between extrinsic (smoking, sedentary lifestyle, poor nutrition) and intrinsic (sex, age, lipid levels, hypertension) factors, each of which may have underlying genetic components, making it difficult to divide CAD risk into genetic and non-genetic factors. However, using these intermediate disease-related intrinsic factors as genetic traits may help to identify novel CAD genetic loci.

We have previously reported a genome-wide linkage scan for early-onset CAD using the GENECARD family-based cohort, which identified nine genomic regions linked to CAD [7]. The 1q and 3q regions have been fine-mapped and the susceptibility genes identified (including *FAM5C *and *KALIRN*, respectively) [8,9]. The signal for 5q31 was present in the overall sample and was not unique to any one phenotypic subset. However, using ordered subset analysis (OSA) to dissect genetic heterogeneity and using lipid levels as quantitative traits, we found that the evidence for linkage on chromosome 5q was increased in families with higher mean total and low density lipoprotein-cholesterol (LDL-C) [10].

Rather than focusing on disease status alone as the trait of interest using one analytic technique, one can apply multiple methods to CAD and disease-related traits within one genomic region, thereby exploring the solution space of the combined analyses and identifying overlapping results; such results act as an internal replication and increase the likelihood that the genetic variant is truly involved in the pathogenesis of CAD. Recently Williams and Haines argued that the replication standard is a strong indicator of a true genetic effect and possibly preferable to the p-value standard [11].

Thus, we report herein our work to fine-map the CAD susceptibility locus on chromosome 5q31, using association analyses of quantitative (LDL-C) and qualitative (CAD and atherosclerosis) traits, using the quantitative results to prioritize the results obtained from qualitative analyses. We conducted this study in several relatively large and independent CAD cohorts, including 1908 individuals from a cohort of patients undergoing cardiac catheterization (CATHGEN), 827 individuals from a family-based study of early-onset CAD (GENECARD) and 162 individuals from a repository of aortic tissue collected from deceased donors. Using this approach of analyses performed in parallel, we identified four genes on chromosome 5q31-33 (*SPOCK1*, *PPP2R2B*, *PRELID2*, and *EBF1*) as candidate susceptibility genes for CAD mediated through LDL-C.

## Methods

### Study populations

All subjects signed a current informed consent form and these studies were approved by the institutional review boards of each participating center.

#### GENECARD family-based study of early-onset CAD

*Genetics of Early Onset Cardiovascular Disease (*GENECARD) is a multicenter family-based linkage study of early-onset CAD using an affected sibling pair based approach; study methods have been described [7]. For GENECARD, early-onset CAD was defined as: MI or unstable angina, coronary angiography showing at least 50% stenosis in a major vessel, revascularization procedure as either percutaneous coronary intervention or coronary artery bypass graft, or a functional test showing reversible myocardial ischemia, occurring before the age of 51 in men and before the age of 56 in women. Of the 438 families included in the original linkage study [7], we selected 254 families, including 726 individuals (504 affected and 222 unaffected) for analysis of cardiovascular endpoints. These families were selected based on the availability of an unaffected family member to maximize power for association analyses (151 families). In addition, families identified from OSA [10] that contributed to the linkage peak on chromosome 5 were also included (103 families). For the analysis of LDL cholesterol traits, 827 individuals from these 254 families were used. LDL-C values were either extracted from medical records or directly measured using the Boehringer Manheim cholesterol enzymatic kit (Roche Diagnostics, Indianapolis, IN, USA) as previously detailed [10]. Given that LDL-C measurements derived from medical records were estimated using the Friedewald equation [12], any individual with triglyceride levels greater than 400 mg/dL were coded as missing for LDL-C. LDL-C measurements greater than four standard deviations from the mean were coded as missing in order to exclude undue influences of extreme outliers.

#### CATHGEN non-familial cohort

The CATHGEN biorepository consists of sequential individuals recruited through the cardiac catheterization laboratory at Duke University Hospital (Durham, NC). Case-control status in the CATHGEN sample was assigned as a function of coronary artery disease index (CADi) and age, such that controls had reached a sufficient age to be at risk of developing disease [9]. All CATHGEN subjects fasted for a minimum of seven hours prior to blood sample collection. Blood was collected via the femoral artery and processed immediately for collection of plasma, and then frozen within hours at -80°C. For this study, we selected 1908 CATHGEN subjects based on their CAD status as previously used for genetic analyses [9]. We had two sources of LDL-C levels; derived from the medical records for a subset of the participants; and lipoprotein particle number concentration measured in stored, frozen, fasting plasma by nuclear magnetic resonance spectroscopy through Liposcience (Liposcience, Raleigh, NC, USA), using published techniques [13,14]. For quantitative trait analyses, total LDL particle number (LDLP) was used as a surrogate for LDL-C; in those CATHGEN individuals for whom both LDL-C and LDLP levels were available (N = 669), the two measures were strongly correlated (r = 0.67, p < 0.0001). Leptin levels were available for 380 individuals from the CATHGEN sample, as previously reported [10,15]

#### Human Aorta Tissue Collection

A collection of 162 aortas were harvested from deceased donors and prepared as previously described [16]. DNA was extracted from the tissue for genotyping using standard protocols. In addition, histopathological studies of the aortas were performed. Specifically, samples were assessed for extent of early atherosclerotic lesions with Sudan IV staining and severe disease assessed by the extent of raised lesions. The burden of atherosclerosis in the aortas was measured using the protocol described in the Pathobiological Determinants of Atherosclerosis in the Young study (PDAY) and were given a graded score (1-4) [17]. As these aortas were harvested from deceased donors, the clinical information attached to each sample was limited, consisting of sex, age, and race.

### Laboratory Methods

#### SNP Selection

SNPs were selected for genotyping based on both the physical distance between SNPs (density dependent selection) and based on the pattern of linkage disequilibrium (LD) within the region (tagging SNPs). SNP map positions and gene identities were derived from the most recent draft of the human genome available (GRCh37/hg19). Within the region of highest lod scores, the average SNP spacing was 1 per 20 kb. Within the flanking genomic regions, SNPs were selected for an average density of 1 per 50 kb. In addition tagging SNPs were selected to capture LD information within coding regions using HapMap data and the Tagger algorithm with the following criteria: an r-squared greater than or equal to 0.7 and a minor allele frequency (MAF) greater than or equal to 0.05; LD between SNPs was visualized using Haploview [18]. One SNP was chosen for each LD bin. Priority was given to coding SNPs, followed by SNPs within known regulatory regions, intronic SNPs, and SNPs located within the 5' or 3' UTR, resulting in a list of 744 haplotype tagging SNPs with one SNP per LD bin. Finally, any SNPs with an Illumina score of less than 0.6, a MAF of less than 0.05, or coded as a potential failure by the Illumina software were excluded from the selection. The final list contained 2,256 SNPs, composed of both density-dependent (1,512) and tagging (744) SNPs.

#### Genotyping

Genomic DNA from the GENECARD and CATHGEN samples was extracted from whole blood using the PureGene system (Gentra Systems, Minneapolis, Minnesota, USA). SNPs were genotyped in two rounds, initially at the Center for Human Genetics at Duke University and subsequently through the NHLBI funded Seattle SNPs (http://pga.gs.washington.edu). The genotyping was performed using the Illumina GoldenGate technology (San Diego, CA, USA). To ensure genotyping accuracy and reliability, several quality control methods were used including two HapMap CEPH individuals and two duplicate individuals included per 96-well plate. SNPs with call rates less than 95% (N = 174) were excluded and individuals with a less than 90% genotyping rate were excluded (N = 57), resulting in 2082 SNPs on a total of 2823 individuals available for analysis. Of those, 20 deviated significantly from Hardy-Weinberg equilibrium (HWE) in Caucasians (p < 0.001) [19]. These 20 SNPs were analyzed, as it has been shown that some deviations from HWE are consistent with reasonable models for complex disease [20]. However, none of these 20 SNPs was significant in any of the analyses performed and had no impact on the reported results.

### Statistical Analyses

#### Association with Quantitative LDL-cholesterol Traits

In the GENECARD sample, association between each individual SNP with LDL-C was performed using the quantitative disequilibrium test (QTDT) [21] and a linear model. Of the available GENECARD sample, an average of 122 trios was analyzed per marker (range 30-196, median 130). In the CATHGEN sample, genotypic and allelic associations between each individual SNP and LDLP were assessed using multivariable logistic regression adjusted for race, age, and sex. Given the low power for association in the GENECARD study, we chose to combine the quantitative analysis results for each SNP from GENECARD and CATHGEN using Fisher's method for combining p-values. Given previous reports that one of our identified genes (*EBF1*) is associated with decreased leptin levels in a murine knockout model [22], we used the Wilcoxon rank sum test to test for association of SNPs within and flanking *EBF1 *with leptin levels in those CATHGEN individuals with available leptin data (N = 380).

#### Association/Linkage Analysis with Cardiovascular Disease

The total SNP panel was tested for association with CAD in GENECARD and CATHGEN and with atherosclerosis in the aorta samples. In the GENECARD study, parametric two-point linkage for early-onset CAD was performed with a recessive (at risk allele freq 0.20, and penetrance 0.001) and dominant (at risk allele freq 0.01, and penetrance 0.001) model using Vitesse [23] and Homog [24]. These tests were conducted to provide independent validation of SNPs showing evidence for association. In the presence of association and linkage, there tends to be a positive correlation between tests of association and linkage; however, there is no such correlation between tests when linkage and association are not present, implying that true positive results in one test tend to be reflected by positive results in another [25]. Family based association with early onset CAD was performed using the association in the presence of linkage test (APL) [26]. This test appropriately accounts for the non-independence of affected siblings and calculates a robust estimate of the genetic variance.

In the CATHGEN cohort, we used multivariable logistic regression adjusted for race and sex, using allelic and genotypic models to test for association with CAD case-control status. In addition, a second CAD case-control series was constructed using the subset of the GENECARD probands (N = 150) that were sampled from North America to more closely resemble the CATHGEN controls (N = 400), as we have previously done [9]; in these analyses, logistic regression was used to test for association between individual SNPs and CAD adjusted for race and sex.

In the aorta sample, the qualitative phenotype was atherosclerosis status, defined by a histopathologic index of atherosclerosis [9], which was analyzed via multivariable linear regression, with adjustments for race and sex under genotypic and allelic models. For all analyses, any results with a nominal p-value ≤ 0.05 were considered to be significant. We did not specifically adjust for multiple comparisons, as our method of comparing multiple related analyses in independent datasets, which we refer to as analyses in parallel, provides internal replication for significant results in the same gene in multiple analyses and further support the significance of the initial observation. Analyses were conducted using SAS Version 9.1 (Cary, NC) unless otherwise specified for specific statistical analysis programs (i.e. QTDT, APL and linkage).

## Results

### Quantitative Trait Associations for LDL-cholesterol Traits

**Figure 1:**
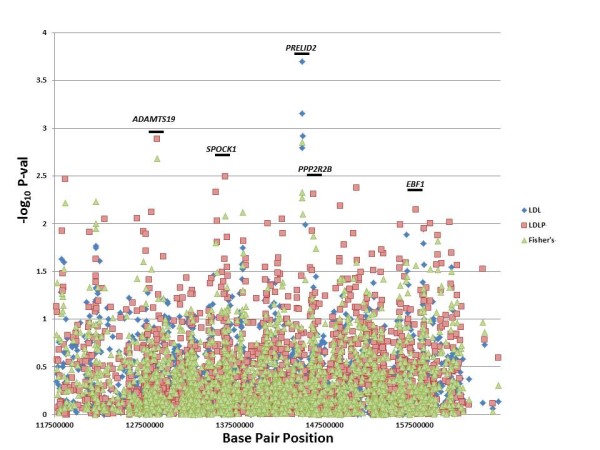
**Quantitative Associations by Base Pair Position**. This figure displays the results of association of SNPs with LDL cholesterol traits in the GENECARD, CATHGEN and aorta samples, with -log10 of the p-value (Y-axis) versus the base pair position of the SNP (X-axis). The five candidate genes are labeled with their approximate positions indicated by a horizontal bar.

The baseline characteristics of the overall study populations have been reported [9]. LDL-C measurements were available for 827 individuals in the GENECARD cohort, with a mean LDL-C concentration of 127.1 mg/dL (standard deviation [SD] 52.3 mg/dL). Family-based association resulted in 32 SNPs in 17 distinct genes that were significantly associated with LDL-C (Additional File 1 Table S1), with the four most significant SNPs residing in the gene *PRELID2 *, (PRELI domain containing 2 isoform) (rs10074645, rs6893183, rs17103583, and rs1865009, p = 0.0002-0.002), which were all in linkage disequilibrium with each other (D' ranging from 0.85-0.95). In the CATHGEN cohort (N = 1,908), mean LDLP levels were 1,131 nmol/L (SD 413 nmol/L). In CATHGEN, we found 102 SNPs in 46 distinct genes that were significantly associated with LDLP levels (Additional File 2 Table S1), with the most significant findings for SNPs in the genes *SPOCK1 *(sparc/osteonectin, cwcv and kazal-like domains) (rs17600115, p = 0.003) and *PPP2R2B *(phosphatase 2 regulatory subunit B family) (rs2125443, p = 0.005). Given low power for association in the GENECARD study, the p-values from the GENECARD and CATHGEN studies were combined using Fisher's method, resulting in 51 SNPs with combined p-values ≤ 0.05 (Table 1). As such, we found ten genes with significant results for association with LDL-C phenotypes in both the GENECARD and CATHGEN cohorts, with the strongest, most consistent results for SNPs in *PRELID2*, *SPOCK1*, *PPP2R2B*, and *EBF1 *(Early B-Cell Factor 1). The results for the quantitative analyses with LDL-C traits are summarized in Figure 1.

**Table 1:** Association results for chromosome 5q31 SNPs with LDL-cholesterol traits in the combined GENECARD and CATHGEN cohorts.

SNP	Gene	Physical Location (bp)	P-value
**rs10074645**	***PRELID2***	**144965385**	**0.001**
rs1895172	*ADAMTS19*	128874733	0.002
**rs6893183**	***PRELID2***	**145018484**	**0.005**
**rs1865009**	***PRELID2***	**144989633**	**0.01**
rs1460038	*intergenic*	122077475	0.01
rs32652	*TNFAIP8*	118705545	0.01
rs11957633	*SIL1*	138364394	0.01
**rs17103583**	***PRELID2***	**145044493**	**0.01**
**rs17600115**	***SPOCK1***	**136420952**	**0.01**
rs7727137	*intergenic*	122081840	0.01
rs1460039	*RP11-166A12.1*	122051006	0.01
**rs2125443**	***PPP2R2B***	**146229428**	**0.01**
rs4921307	*ATP10B*	160026777	0.02
rs1558095	*intergenic*	135429640	0.02
rs1558095	*intergenic*	135429640	0.02
rs728937	*intergenic*	146485682	0.02
rs7721110	*SIL1*	138482506	0.02
rs17164449	*CTC-228N24.3*	127342272	0.02
rs2304052	*SPARC*	151054227	0.03
rs246869	*FAM71B*	156584384	0.03
rs44156	*intergenic*	157625206	0.03
rs6596460	*SIL1*	138414180	0.03
rs876600	*SLC27A6*	128235848	0.03
rs6595178	*DMXL1*	118532034	0.03
rs6863332	*intergenic*	159234840	0.03
rs962271	*ATP10B*	160054549	0.03
rs418210	*GABRG2*	161580983	0.03
rs889010	*TRPC7*	135572910	0.03
rs367153	*ITK*	156610846	0.04
rs383915	*SLC36A2*	150695724	0.04
rs35525	*MEGF10*	126699347	0.04
rs10875552	*PPARGC1B*	149189489	0.04
rs12374480	*intergenic*	154001174	0.04
rs2240793	*SLC6A7*	149583300	0.04
rs4913054	*SH3RF2*	145339561	0.04
rs880770	*PPARGC1B*	149154835	0.04
rs1036199	*HAVCR2*	156531736	0.04
rs13182800	*NR3C1*	142801480	0.04
rs12188371	*intergenic*	117764534	0.04
rs6556615	*SGCD*	155859368	0.04
rs938537	*intergenic*	160458036	0.04
rs4244032	*NR3C1*	142794725	0.04
rs1350375	*intergenic*	161358888	0.05
rs6595416	*SNX24*	122268004	0.05
rs1800449	*SRFBP1*	121413208	0.05
**rs2950952**	***SPOCK1***	**136654594**	**0.05**
rs193730	*intergenic*	141154583	0.05
rs162486	*intergenic*	123073478	0.05
rs586115	*FBN2*	127662875	0.05
**rs17717527**	***EBF1***	**158466283**	**0.05**
rs1465689	*ARSI*	149685855	0.05

The 51 SNPs with p-values ≤0.05 using Fisher's method for combining results from CATHGEN and GENECARD are presented.. Results for the four candidate genes (*PPP2R2B, EBF1, SPOCK1*, and *PRELID2*) are in bold.

Given previous studies showing that mouse knockout models for EBF1 have reduced leptin levels [22], we also tested all *EBF1 *SNPs and SNPs upstream and downstream of *EBF1 *(N = 78 SNPs) for association with leptin levels (median 13.8 micrograms/L, range 0.4-104.9 micrograms/L) in a subset of CATHGEN (N = 380); six *EBF1 *SNPs were nominally significantly associated with leptin levels (Additional File 3 Table S1). The most significant result was for rs13165442 (p = 0.001), however that SNP was not significant for association with lipid levels or CAD. The SNP rs17635991 in *EBF1*, however, was nominally associated with CAD (p = 0.02) and with leptin levels (p = 0.03).

### Qualitative Trait Association with CAD/atherosclerosis

Those genes with significant results for association with LDL-C traits were retained for comparison to the CAD endpoint results; only those genes which had a significant result in at least one CAD endpoint were retained for analysis. This list was further reduced by selecting those genes with the lowest p-values and the most consistent results across all analyses (i.e. significant results for the largest number of independent tests).

In the GENECARD sample, 102 SNPs in 45 distinct genes were significantly associated with CAD using APL (Additional File 4 Table S1); the most significant results were SNPs in the genes *PPP2R2B *(rs7736604, p = 0.0003) and *SPOCK1 *(rs17170899, p = 0.003). Two-point linkage analysis in GENECARD resulted in 16 SNPs in seven genes with LOD scores ≥ 1.5 at θ = 0, with the highest LOD scores of 2.1 obtained for two SNPs (rs17166444 in the *FSTL4 *(follistatin-like 4 precursor) and rs7736046 in *PRELID2*) (Additional File 5 Table S1) and evidence for linkage at both *SPOCK1 *(rs1919515, LOD = 1.8) and *PRELID2 *(rs7736046, LOD = 2.1).

**Figure 2:**
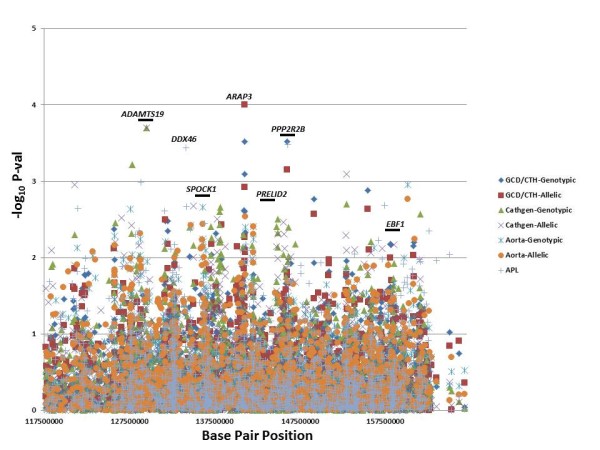
**Qualitative Associations by Base Pair Position**. This figure displays the results of association of SNPs with CAD/atherosclerosis in the GENECARD, CATHGEN and aorta samples, with -log10 of the p-value (Y-axis) versus the base pair position of the SNP (X-axis). The five candidate genes are labeled with their approximate positions indicated by a horizontal bar; other select genes with significant results are labeled and have no horizontal bar.

In the CATHGEN sample, we found 178 SNPs in 58 genes that were associated with CAD (Additional File 6 Table S1) with the most significant results for an intergenic SNP (rs10050603, p = 0.0002) 628 kb distal to *EBF1*. In addition, there were significant results for *PPP2R2B *(rs1383169, p = 0.009), *PRELID2 *(rs7713855, p = 0.003), *SPOCK1 *(rs6872714, p = 0.02), and *EBF1 *(rs6865969, p = 0.007). The GENECARD proband-CATHGEN case/control validation cohort analysis resulted in 132 significant SNPs in 60 distinct genes (Additional File 7 Table S1), with the most significant evidence for association with CAD for SNPs in *ARAP3 *(ArfGAP with RhoGAP domain, ankyrin repeat and PH) (rs6895094, p = 0.0001) and the transcript CTB-99A3.1 (rs11744339, p = 0.0003), which overlaps the genomic location *of PPP2R2B*. In addition, the genes *EBF1 *(rs17712788, p = 0.007), *PPP2R2B *(rs1383169, p = 0.01), and *SPOCK1 *(rs6884385, p = 0.01) also had significant associations. In the aorta samples, we found 165 SNPs in 49 genes (Additional File 8 Table S1) that were significantly associated with the degree of atherosclerosis. The most significant association was with a SNP in the gene ATPase, class V, type 10B *(ATP10B *(; rs1990889, p = 0.001). The genes *SPOCK1 *(rs2043478, p = 0.008), *PPP2R2B *(rs1383167, p = 0.02), and *EBF1 *(rs6865969, p = 0.02) also contained significantly associated SNPs. All of the qualitative results for association with CAD or degree of atherosclerosis are summarized in Figure 2.

### Analyses in Parallel

**Figure 3:**
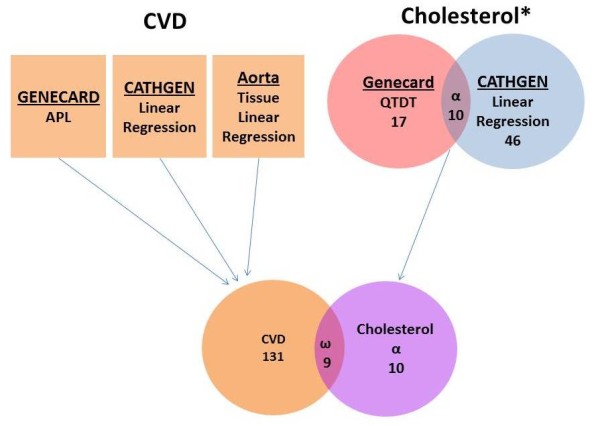
**Analytical Strategy for 'Parallel Analysis' of Chromosome 5q31 Region**. This figure details the study design, cardiovascular cohorts and analytic techniques used, and the number of unique genes containing SNPs with significant associations (indicated underneath the method used). The union of quantitative results (α) indicates the subset of genes shared by the two methods. The qualitative trait (CVD) was analyzed in GENECARD, CATHGEN, and aortas and the total number of unique genes containing a significant SNP in any of those analyses is indicated. The commonality of the genes between the quantitative and qualitative analyses (N = 9, *PRELID2*, *SPOCK1, EBF1*, *PPP2R2B*, *DMXL1*, *DTWD2*, *GABRG2*, *GLRA1*, and *RP11-166A12.1*) is indicated by ω.

Of the 51 SNPs in ten genes that were significantly associated with LDL-C traits, we further reduced this list of candidates by selecting only those genes which had at least one significant association with any of the CAD endpoints, resulting in a list of nine genes (Figure 3). These genes were then prioritized by both the size of the p-value and consistency in effect across both quantitative and qualitative analyses. This selection resulted in a final list of four genes (Table 2) with the most consistent association with LDL-C traits and CAD/atherosclerosis, although with varying effects for SNPs within those genes. The LD pattern for significant SNPs within each gene showed few examples of strong LD between significant SNPs (Additional Files 9, 10, 11 and 12), with the exception of *PRELID2 *(Additional File 12). In order to verify the independence of these four loci, pairwise LD was calculated for all SNPs across the four genes; there was no LD between SNPs from differing genes (data not shown).

**Table 2:** Summary of results across cohorts for four candidate genes identified through parallel analysis.

Gene	SNP	Quantitative Results			Qualitative Results				
		(LDL Cholesterol Traits)			(CAD, Atherosclerosis)				
		**LDL-GENECARD**	**LDLP-CATHGEN**	**Fisher's Combined**	**GENECARD**	**CATHGEN**	**GENECARD Probands-CATHGEN Controls**	**Aortas**	**Linkage (LOD)**
***PPP2R2B***	**rs10037715**	**0.05**	0.77	0.15	0.82	0.31	0.25	0.68	-17.8
	**rs2125443**	0.38	**0.001**	**0.01**	0.71	0.31	0.84	0.13	-14.4
	**rs7736604**	0.92	0.01	0.06	**0.0003**	0.22	0.45	0.02	-13.2
	**rs160974**	0.92	0.62	0.62	0.47	**0.004**	0.50	0.65	-18.0
	**rs1383169**	0.81	0.11	0.31	0.39	0.01	**0.01**	0.49	-9.3
	**rs1383167**	0.99	0.28	0.63	0.45	0.08	0.92	**0.02**	-14.2
***EBF1***	**rs17717527**	**0.01**	0.73	**0.05**	0.40	0.55	0.13	0.31	-19.5
	**rs6865969**	0.84	**0.01**	0.05	0.40	**0.01**	0.13	**0.02**	-20.0
	**rs4704963**	0.78	0.24	0.51	**0.03**	0.67	0.38	0.46	-6.4
	**rs17712788**	0.08	0.90	0.90	0.85	0.33	**0.01**	0.16	-8.3
***SPOCK1***	**rs10054991**	**0.05**	0.84	0.17	0.72	0.79	0.14	0.33	-14.8
	**rs17600115**	0.33	**0.003**	**0.001**	0.85	0.27	0.43	0.05	-5.7
	**rs17170899**	0.71	0.98	0.95	**0.004**	0.63	0.82	0.86	-1.6
	**rs6872714**	0.19	0.80	0.44	0.91	**0.02**	0.64	0.04	-16.8
	**rs6884385**	0.16	0.17	0.13	0.86	0.06	**0.02**	0.24	-16.0
	**rs2043478**	0.88	0.77	0.94	0.49	0.33	0.23	**0.01**	-17.3
	**rs1919515**	0.80	0.59	0.82	0.42	0.74	0.05	0.36	**1.8**
***PRELID2***	**rs10074645**	**0.0002**	0.71	**0.001**	0.93	0.67	0.19	0.66	-2.7
	**rs11950106**	0.73	**0.04**	0.12	0.56	0.09	0.30	0.73	-13.7
	**rs7713855**	0.93	0.54	0.85	0.77	**0.003**	0.13	0.33	-16.2
	**rs13179436**	0.65	0.77	0.85	0.52	0.005	**0.03**	0.26	-16.0
	**rs443033**	0.50	0.90	0.81	0.48	0.05	0.14	**0.04**	-10.1
	**rs7736046**	0.98	0.80	0.98	0.86	0.03	0.81	0.19	**2.1**

P-values for the most significant results for SNPs for each analysis for the four top candidate genes (*PPP2R2B, EBF1, SPOCK1, and PRELID2*) are presented. The most significant result for each gene and analysis is in bold.

## Discussion

We have demonstrated herein that disease-related intermediate traits can identify novel disease risk genes. Specifically, we used LDL-cholesterol traits to fine-map a linkage peak on chromosome 5 from the GENECARD study of early-onset CAD with integration of these results with association and linkage to cardiovascular disease. Using this approach, we have identified four candidate genes (*EBF1*, *PPP2R2B, PRELID2*, and *SPOCK1*) that may represent novel cardiovascular disease risk genes mediated through LDL cholesterol pathways.

Although no genome scans for CAD or MI have reported linkage to this region, there are several potentially related phenotypes that have been mapped to the 5q31 locus including inflammatory or autoimmune conditions (celiac disease [27], asthma [28], Grave's disease [29], psoriasis [30], and Crohn disease [31]) as well as cardiac and vascular phenotypes (cardiomyopathy [32], intracranial aneurysm [33], infantile hemangioma [34], and systolic and diastolic blood pressures [35,36]). Several GWAS and meta-analyses have been published for lipid related traits [37-40]. However, only one study has reported a significant association for any genes on chromosome 5q31-33, for the gene T-cell immunoglobulin and mucin domain containing 4 (*TIMD4*) [38], which is 1.7 Mb centromeric to *EBF1 *and 9.9 Mb telomeric to *PPP2R2B*. Nominally significant results were obtained for SNPs within *TIMD4 *and its ligand (*HAVCR2*), which is 152 kb telomeric to *TIMD4 *(Additional Files 2 Table S1, 6 Table S1, and 8 Table S1).

Using a database of publicly available GWAS results from NHGRI, we looked for any reports of significant associations for SNPs within the 1 lod interval on chromosome 5. There are few references to cardiovascular disease or disease-related traits. However, none of these SNPs overlap with significant SNPs in our results, with the exception of two studies of hypertension/systolic blood pressure [41,42], each of which reported one significant association with a SNP in *EBF1*; unfortunately, neither of those two SNPs was examined in our study.

The gene *EBF1 *is involved in hematopoiesis and immunity [43]. Interestingly, studies of knockout mice have identified a role for *EBF1 *in metabolism [22], a cardiovascular disease related phenotype. The null mice described by Fretz et al. have a unique metabolic syndrome characterized by lipodystrophy, hypotryglyceridemia, and hypoglycemia, while having an increased metabolic rate and decreased leptin levels. The mouse lipodystrophy is characterized by an increase in yellow adipose tissue in bone marrow and a marked decrease in white adipose tissue (by as much as 90%), relative to wild type controls. These findings are consistent with *EBF1*'s regulation of adipocyte progenitors [44,45]. In our study, SNPs in *EBF1 *were significantly associated with LDL cholesterol traits and the CAD endpoints, with the exception of two-point linkage. In addition, *EBF1 *variation was associated with leptin levels in our sample, although the results for individual SNPs were inconsistent with their association with lipids and CAD endpoints. This may suggest that *EBF1 *has a similar role in regulating adiposity and lipid metabolism in humans, and that variants in the gene may represent good candidate polymorphisms for cardiovascular disease and dyslipidemia in humans.

Of the other candidate genes identified, *SPOCK1 *is associated with age at menarche via a genome-wide association study [46,47]. *SPOCK1 *encodes a proteoglycan that functions as a protease inhibitor; although initially identified in testes [48], it is expressed in many human tissues including blood. SNPs within *SPOCK1 *were tested for sex-specific effects in our sample via a stratified analysis; no significant sex effects were observed (data not shown). *PPP2R2B *encodes a brain specific regulatory subunit of a protein phosphatase and is the causal locus for a Mendelian disease, a form of spinocerebellar ataxia (SCA12, OMIM# 604326). Little is known about the function of the gene *PRELID2 *other than it contains a 'prel-like domain,' from which its name is derived.

Using a strategy of analyses in parallel we have identified four novel candidate genes for cardiovascular disease. The ability to reduce the list of potential candidates within the linkage region on chromosome 5q31-33 from a few hundred to only four is proof of principle that this strategy may be a useful tool for analyzing complex traits. In addition, had we relied upon CAD endpoint analyses alone, we would have obtained less significant associations overall and would have prioritized a different set of candidate genes. One of the major strengths of our study is the detailed phenotype information available for both the GENECARD cohort and CATHGEN biorepository. The rigorous inclusion criteria and case definitions used in GENECARD and CATHGEN have led to objective measures for CAD endpoints, a phenotype that would otherwise have a subjective definition. In this particular study, direct sequencing, rather than SNP genotyping across multiple samples, would not have been appropriate; if we were to re-sequence a sub-set of the sample, it is not clear which individuals would be selected for such sequencing, particularly for the continuous quantitative traits we examine. Finally, our sample is of mixed ethnicity (Caucasian and African-American), which would necessitate a separate re-sequencing effort for each ethnicity.

This study has some limitations. First, all four genes may be CAD susceptibility genes and act independently (as the LD patterns in our samples suggest). However, it is possible that SNPs within our sample are in LD with causal SNPs at another locus and the association results from these four genes may not be independent. Second, we have focused on the consistency of the results at the gene level (i.e., which genes have SNPs that are significant in multiple analyses). However, it is not the case that the same SNPs are significant in those analyses or, in the case that the same SNP is significant, that the magnitude of that significance is similar between analyses. Thus, we cannot begin to identify individual SNPs within a candidate gene that are likely to be driving the results via direct or indirect biological action. This can be explained, in part, by the fact that the phenotypes, while correlated, are not perfectly correlated. Therefore, it is expected that there will be differences in the p-values for associations with different phenotypes and this could cause certain associations to fall outside the nominal p-value cutoff. Finally, the results were not interpreted in the context of correction for multiple comparisons. There are two main difficulties with applying such corrections to these results, i.e. a Bonferroni correction which would be overly conservative. First, the phenotypes examined are correlated, therefore the analyses conducted using more than one phenotype within the same sample are not independent. Second, if we look at the results sequentially, with one analysis conducted after another, then the prior probability that a given SNP in a gene of interest will be significant in subsequent analyses is non-negligible. We are not relying upon the magnitude of any given p-value to identify a single gene in the region as the most likely to explain the original evidence for linkage. Rather, we are suggesting that a set of genes be examined as likely candidate susceptibility loci for cardiovascular disease that is mediated by lipid levels.

In order to identify which gene or genes among the four we have selected contains variation for cardiovascular disease mediated by LDL cholesterol pathways, there are several methodologies available. Re-sequencing studies could be conducted in our sample, either in the entire population or by using individuals with extreme trait values (i.e., very high/low LDL-C levels or very early onset cardiovascular disease), as these data would capture all of the variation present in those samples and not rely upon common variants identified through a different, although ethnically similar, sample (i.e., CEPH Caucasians). In addition, those genes for which the biological function is known (i.e., *EBF1*) could have their level of activity or functionality directly assessed in genotyped samples. Such an approach can identify subsets of variation that appear to have functional consequences. However, due to LD within the variants, such results can still be ambiguous, in which case promoter and gene constructs can be created and assayed in the laboratory, allowing one to query the functional consequences of individual variants

## Conclusions

In summary, we propose a strategy of parallel analysis where results from analyses of disease-related intermediate traits for a complex disease can be considered jointly with qualitative results from mapping of the disease trait itself, potentially enabling discovery of novel disease genes mediated through these intermediate phenotypes that might not have been identified using disease status alone. In addition, this strategy may allow the dissection of genetic heterogeneity mediated through the intermediate phenotype. We have applied this strategy to the fine mapping of a linkage peak for early-onset CAD and thereby have demonstrated replication of four genes within a region on chromosome 5q31. These genes, in particular, *EBF1 *given its potential biological plausibility, serve as novel candidate loci for cardiovascular disease and should be further evaluated.

## Authors' contributions

DN performed the analysis, data interpretation, and authored the manuscript. BS performed genotyping, statistical analysis, data interpretation, and drafted part of the manuscript. CH performed analyses and informatics support. JJ performed analyses. JS performed analyses and informatics support. D Crosslin, D Crossman, MS, CG, DS, and PG performed phenotyping and contributed to the manuscript. WK contributed to the study design and the manuscript. SG contributed to study design and supervised the genotyping. EH contributed to study design and supervised the analysis. SS managed the chr 5. project, conceived of and contributed to the study design, performed phenotyping, supervised the analysis, interpreted the data, edited the manuscript, and is corresponding author. All authors have read and approved this manuscript.

## Supplementary Material

Additional file 1 **Genes associated with LDL cholesterol traits in the GENECARD study**. This table displays minor allele frequencies (MAF), sample sizes, and p-values for the most significant SNPs associated with LDL cholesterol using the quantitative trait disequilibrium test (QTDT) in the GENECARD study. Note that the sample size is less than the overall GENECARD study sample size since not all families could be included due to lack of unaffecteds. SNPs within our four key candidate genes are in bold. Click here for file

Additional file 2 **Genes associated with LDL cholesterol traits in the CATHGEN cohort**. Displayed are the results for association of SNPs with LDLP levels in the CATHGEN sample. The significant SNPs are shown followed by the genic location, base pair position, and the corresponding p-value. SNPs within our four key candidate genes are presented in bold. Click here for file

Additional file 3 **Associations of *EBF1 *SNPs with Leptin Levels**. Six SNPs within and flanking *EBF1 *are listed with their Wilcoxon rank test p-values for association with leptin levels in CATHGEN in the total sample with available leptin levels, and stratified by race. Click here for file

Additional file 4 **Association of SNPs with CAD in the GENECARD cohort**. Displayed are results of the qualitative analysis using APL in the GENECARD sample. All significant SNPs are shown followed by their genic location, base pair position, and their corresponding p-values. Results within our four key candidate genes are displayed in bold. Click here for file

Additional file 5 **Linkage results for SNPs with early-onset CAD in the GENECARD cohort**. Displayed are linkage results for the GENECARD sample. All SNPs with two-point lod scores ≥ 1.5 are listed followed by their genic location, base pair position, individual lod score, and genetic model used. Click here for file

Additional file 6 **Association of SNPs with CAD in the CATHGEN Cohort**. Displayed are all significant results for the case-control analysis of SNPs with CAD in the CATHGEN sample. Each significant SNP is listed followed by their genic location, base pair, p-value, odds ratio, and 95% confidence interval for the genotypic and allelic models, respectively. Click here for file

Additional file 7 **Association of SNPs with CAD for GENECARD proband vs. CATHGEN controls sample set**. Displayed are the results of the GENECARD proband-CATHGEN control analysis, with all significant SNPs listed followed by their genic location, base pair position, p-value, odds ratio, and 95% confidence intervals for the genotypic and allelic models used. Click here for file

Additional file 8 **Association of SNPs with Degree of Atherosclerosis in the Aorta Samples**. Displayed are results of association of SNPs with degree of atherosclerosis in the aorta samples, with each significant SNP listed followed by their genic position, base pair position, p-value, odds ratio, and 95% confidence interval for the genotypic and allelic models. Click here for file

Additional file 9 **Linkage disequilibrium patterns of select SNPs in *PPP2R2B***. LD pattern using Haploview, with r-squared values displayed within each box and the shading of the boxes corresponding do D' values. Click here for file

Additional file 10 **Linkage disequilibrium patterns of select SNPs in *EBF1***. LD pattern using Haploview, with r-squared values displayed within each box and the shading of the boxes corresponding do D' values. Click here for file

Additional file 11 **Linkage disequilibrium patterns of select SNPs in *SPOCK1***. LD pattern using Haploview, with r-squared values displayed within each box and the shading of the boxes corresponding do D' values. Click here for file

Additional file 12 **Linkage disequilibrium patterns of select SNPs in *PRELID2***. LD pattern using Haploview, with r-squared values displayed within each box and the shading of the boxes corresponding do D' values. Click here for file
