# Supplementary material for: Fine mapping of a linkage peak with integration of lipid traits identifies novel coronary artery disease genes on chromosome 5
Source: BMC Genet. 2012 Feb 27;13:12. doi: 10.1186/1471-2156-13-12 (PMC3309961; doi:10.1186/1471-2156-13-12)
Supplement: Additional file 1 — Genes associated with LDL cholesterol traits in the GENECARD study. This table displays minor allele frequencies (MAF), sample sizes, and p-values for the most significant SNPs associated with LDL cholesterol using the quantitative trait disequilibrium test (QTDT) in the GENECARD study. Note that the sample size is less than the overall GENECARD study sample size since not all families could be included due to lack of unaffecteds. SNPs within our four key candidate genes are in bold. [file 1471-2156-13-12-S1.DOCX]

**Additional File 1**

**Table S1. Genes associated with LDL cholesterol traits in the GENECARD study.** This table displays minor allele frequencies (MAF), sample sizes, and p-values for the most significant SNPs associated with LDL cholesterol using the quantitative trait disequilibrium test (QTDT) in the GENECARD study. Note that the sample size is less than the overall GENECARD study sample size since not all families could be included due to lack of unaffecteds. SNPs within our four key candidate genes are in bold.

|  |  |  |  |  |
| --- | --- | --- | --- | --- |
| **SNP** | **Locus** | **Physical Location** | **P-value** | **MAF** |
| **rs10074645** | ***PRELID2*** | **144965385** | **0.0002** | **0.08** |
| **rs6893183** | ***PRELID2*** | **145018484** | **0.001** | **0.05** |
| **rs17103583** | ***PRELID2*** | **145044493** | **0.001** | **0.08** |
| **rs1865009** | ***PRELID2*** | **144989633** | **0.002** | **0.08** |
| rs4913054 | *SH3RF2* | 145339561 | 0.01 | 0.08 |
| rs1460038 | Intergenic | 122077475 | 0.02 | 0.41 |
| rs11957633 | *SIL1* | 138364394 | 0.02 | 0.32 |
| rs2029036 | *DTWD2* | 118275869 | 0.02 | 0.09 |
| rs1422282 | *PRDM6* | 122517754 | 0.02 | 0.29 |
| rs6595178 | *DMXL1* | 118532034 | 0.03 | 0.38 |
| rs2043272 | *SIL1* | 138390631 | 0.03 | 0.36 |
| rs12719518 | *SIL1* | 138340976 | 0.03 | 0.37 |
| rs6596460 | *SIL1* | 138414180 | 0.03 | 0.33 |
| rs7445602 | Intergenic | 135123055 | 0.04 | 0.25 |
| rs728937 | Intergenic | 146485682 | 0.04 | 0.48 |
| rs7717375 | *SIL1* | 138291405 | 0.04 | 0.35 |
| rs1990890 | *SNX24* | 122182970 | 0.05 | 0.31 |
