# Supplementary material for: Fine mapping of a linkage peak with integration of lipid traits identifies novel coronary artery disease genes on chromosome 5
Source: BMC Genet. 2012 Feb 27;13:12. doi: 10.1186/1471-2156-13-12 (PMC3309961; doi:10.1186/1471-2156-13-12)
Supplement: Additional file 2 — Genes associated with LDL cholesterol traits in the CATHGEN cohort. Displayed are the results for association of SNPs with LDLP levels in the CATHGEN sample. The significant SNPs are shown followed by the genic location, base pair position, and the corresponding p-value. SNPs within our four key candidate genes are presented in bold. [file 1471-2156-13-12-S2.DOCX]

**Additional File 2**

**Table S1. Genes associated with LDL cholesterol traits in the CATHGEN cohort.** Displayed are the results for association of SNPs with LDLP levels in the CATHGEN sample. The significant SNPs are shown followed by the genic location, base pair position, and the corresponding p-value. SNPs within our four key candidate genes are presented in bold.

|  |  |  |  |
| --- | --- | --- | --- |
| **SNP** | **Gene** | **Physical Location** | **P-value** |
| rs1895172 | *ADAMTS19* | 128874733 | 0.001 |
| **rs17600115** | ***SPOCK1*** | **136420952** | **0.003** |
| rs32652 | *TNFAIP8* | 118705545 | 0.003 |
| rs2304052 | *SPARC* | 151054227 | 0.004 |
| rs1558095 | *intergenic* | 135429640 | 0.005 |
| rs1558095 | *intergenic* | 135429640 | 0.005 |
| **rs2125443** | ***PPP2R2B*** | **146229428** | **0.005** |
| rs10875552 | *PPARGC1B* | 149189489 | 0.01 |
| rs44156 | *intergenic* | 157625206 | 0.01 |
| rs876600 | *SLC27A6* | 128235848 | 0.01 |
| rs35525 | *MEGF10* | 126699347 | 0.01 |
| rs4244032 | *NR3C1* | 142794725 | 0.01 |
| rs162486 | *intergenic* | 123073478 | 0.01 |
| rs889010 | *TRPC7* | 135572910 | 0.01 |
| rs1350375 | *intergenic* | 161358888 | 0.01 |
| rs6863332 | *intergenic* | 159234840 | 0.01 |
| rs193730 | *intergenic* | 141154583 | 0.01 |
| rs6556615 | *SGCD* | 155859368 | 0.01 |
| **rs6865969** | ***EBF1*** | **158502728** | **0.01** |
| **rs7736604** | ***PPP2R2B*** | **146068662** | **0.01** |
| rs299208 | *DTWD2* | 118325429 | 0.01 |
| rs17164449 | *CTC-228N24.3* | 127342272 | 0.01 |
| rs1800449 | *SRFBP1* | 121413208 | 0.01 |
| rs383915 | *SLC36A2* | 150695724 | 0.01 |
| rs13182800 | *NR3C1* | 142801480 | 0.01 |
| rs3805620 | *FBN2* | 127606864 | 0.01 |
| rs4921307 | *ATP10B* | 160026777 | 0.01 |
| **rs2950952** | ***SPOCK1*** | **136654594** | **0.01** |
| rs7721110 | *SIL1* | 138482506 | 0.02 |
| rs2287746 | *TRPC7* | 135548037 | 0.02 |
| rs7701427 | *CAMK2A* | 149632955 | 0.02 |
| rs6580076 | *GALNT10* | 153783753 | 0.02 |
| rs586115 | *FBN2* | 127662875 | 0.02 |
| rs418210 | *GABRG2* | 161580983 | 0.02 |
| rs12374480 | *intergenic* | 154001174 | 0.02 |
| rs17114459 | *intergenic* | 152732763 | 0.02 |
| rs962271 | *ATP10B* | 160054549 | 0.02 |
| rs6861367 | *intergenic* | 129545485 | 0.02 |
| rs26698 | *ARHGAP26* | 142325554 | 0.02 |
| rs4835728 | *DNAJC18* | 138754741 | 0.02 |
| rs880770 | *PPARGC1B* | 149154835 | 0.02 |
| rs17636934 | *SGCD* | 155796872 | 0.02 |
| **rs718703** | ***SPOCK1*** | **136403289** | **0.02** |
| rs1460039 | *RP11-166A12.1* | 122051006 | 0.02 |
| rs2915826 | *ATOX1* | 151119963 | 0.02 |
| rs10875551 | *PPARGC1B* | 149173134 | 0.02 |
| rs1036199 | *HAVCR2* | 156531736 | 0.03 |
| rs185200 | *ARHGAP26* | 142254679 | 0.03 |
| rs938537 | *intergenic* | 160458036 | 0.03 |
| rs1582417 | *intergenic* | 159897501 | 0.03 |
| rs2687527 | *intergenic* | 162165571 | 0.03 |
| rs4365836 | *AC135457.1* | 138732103 | 0.03 |
| rs370479 | *DIAPH1* | 140924806 | 0.03 |
| **rs916852** | ***SPOCK1*** | **136636545** | **0.03** |
| rs7709485 | *TCERG1* | 145894896 | 0.03 |
| rs140616 | *SGCD* | 155810954 | 0.03 |
| rs1030154 | *intergenic* | 165051330 | 0.03 |
| rs17114771 | *GRIA1* | 152915962 | 0.03 |
| **rs2120569** | ***PPP2R2B*** | **146073871** | **0.03** |
| rs4958729 | *GALNT10* | 153718868 | 0.03 |
| rs13184710 | *CTB-1I21.1* | 136089919 | 0.03 |
| rs29843 | *intergenic110* | 151502574 | 0.03 |
| rs23282 | *ARHGAP26* | 142270301 | 0.03 |
| rs6595174 | *DMXL1* | 118440675 | 0.03 |
| rs258768 | *ARHGAP26* | 142561294 | 0.03 |
| **rs12655224** | ***SPOCK1*** | **136730050** | **0.04** |
| rs4836196 | *RP11-166A12.1* | 122064421 | 0.04 |
| **rs11950106** | ***PRELID2*** | **144923237** | **0.04** |
| rs6873053 | *TIMD4* | 156376703 | 0.04 |
| rs256869 | *SEPT8* | 132135372 | 0.04 |
| rs1428155 | *GLRA1* | 151281633 | 0.04 |
| rs2547 | *CTC-321K16.1* | 134907552 | 0.04 |
| rs1105168 | *FAT2* | 150886882 | 0.04 |
| rs2240793 | *SLC6A7* | 149583300 | 0.04 |
| rs252155 | *RBM22* | 150065544 | 0.04 |
| rs4355569 | *CTC-573M9.1* | 128008356 | 0.04 |
| rs33409 | *SYNPO* | 150038265 | 0.04 |
| rs251021 | *DIAPH1* | 140899268 | 0.04 |
| rs712166 | *ARHGAP26* | 142298353 | 0.04 |
| rs919260 | *intergenic* | 151317924 | 0.04 |
| rs6861657 | *intergenic* | 136254338 | 0.04 |
| rs10900864 | *DNAJC18* | 138773090 | 0.04 |
| rs1460038 | *intergenic* | 122077475 | 0.04 |
| rs7732671 | *PPARGC1B* | 149212243 | 0.04 |
| rs251574 | *intergenic* | 134805653 | 0.04 |
| rs210989 | *GABRG2* | 161533712 | 0.04 |
| rs2043280 | *NDFIP1* | 141513691 | 0.04 |
| rs449454 | *NDFIP1* | 141533062 | 0.04 |
| rs7734532 | *DMXL1* | 118484804 | 0.05 |
| rs1465690 | *FAT2* | 150946966 | 0.05 |
| rs11745351 | *intergenic* | 136021073 | 0.05 |
| rs1833754 | *intergenic* | 158818927 | 0.05 |
| rs411219 | *intergenic* | 131451633 | 0.05 |
| rs401750 | *GABRG2* | 161582661 | 0.05 |
| rs6595440 | *CEP120* | 122718736 | 0.05 |
| rs13168599 | *SLC27A6* | 128059350 | 0.05 |
| rs4705336 | *PCYOX1L* | 148747902 | 0.05 |
| rs6888154 | *intergenic* | 150260382 | 0.05 |
| **rs13170526** | ***EBF1*** | **158175669** | **0.05** |
| rs25872 | *FSTL4* | 132785577 | 0.05 |
| rs1469072 | *intergenic* | 144426169 | 0.05 |
| rs7702030 | *intergenic* | 160406428 | 0.05 |
