# Supplementary material for: Fine mapping of a linkage peak with integration of lipid traits identifies novel coronary artery disease genes on chromosome 5
Source: BMC Genet. 2012 Feb 27;13:12. doi: 10.1186/1471-2156-13-12 (PMC3309961; doi:10.1186/1471-2156-13-12)
Supplement: Additional file 3 — Associations of EBF1 SNPs with Leptin Levels. Six SNPs within and flanking EBF1 are listed with their Wilcoxon rank test p-values for association with leptin levels in CATHGEN in the total sample with available leptin levels, and stratified by race. [file 1471-2156-13-12-S3.DOCX]

**Additional File 3**

**Table S1. Associations of *EBF1* SNPs with Leptin Levels**. Six SNPs within and flanking *EBF1* are listed with their Wilcoxon rank test p-values for association with leptin levels in CATHGEN in the total sample with available leptin levels, and stratified by race.

| **SNP** |  | **Overall (N=380)** | **Caucasian**  **(N=268)** | **African- American**  **(N=89)** |
| --- | --- | --- | --- | --- |
| rS1173468 | | 0.01 | 0.04 | 0.03 |
| rS716911 |  | 0.02 | 0.08 | 0.40 |
| rS13163958 | | 0.03 | 0.03 | 0.63 |
| rS13165442 | | 0.001 | 0.03 | 0.04 |
| rS17635991 | | 0.03 | 0.05 | 0.72 |
| rS17056301 | | 0.05 | 0.03 | 0.91 |
