# Supplementary material for: Fine mapping of a linkage peak with integration of lipid traits identifies novel coronary artery disease genes on chromosome 5
Source: BMC Genet. 2012 Feb 27;13:12. doi: 10.1186/1471-2156-13-12 (PMC3309961; doi:10.1186/1471-2156-13-12)
Supplement: Additional file 4 — Association of SNPs with CAD in the GENECARD cohort. Displayed are results of the qualitative analysis using APL in the GENECARD sample. All significant SNPs are shown followed by their genic location, base pair position, and their corresponding p-values. Results within our four key candidate genes are displayed in bold. [file 1471-2156-13-12-S4.DOCX]

**Additional File 4**

**Table S1. Association of SNPs with CAD in the GENECARD cohort.** Displayed are results of the qualitative analysis using APL in the GENECARD sample. All significant SNPs are shown followed by their genic location, base pair position, and their corresponding p-values. Results within our four key candidate genes are displayed in bold.

|  |  |  |  |
| --- | --- | --- | --- |
| **SNP** | **Gene** | **Physical Location** | **CVD-GENECARD** |
| **rs7736604** | ***PPP2R2B*** | **146068662** | **0.0003** |
| rs254711 | *DDX46* | 134140839 | 0.0004 |
| rs1895172 | *ADAMTS19* | 128874733 | 0.001 |
| rs3095710 | *intergenic* | 157394806 | 0.002 |
| rs7717860 | *DDX46* | 134190225 | 0.002 |
| rs919242 | *ZNF474* | 121495859 | 0.002 |
| rs273909 | *SLC22A4* | 131667353 | 0.002 |
| **rs17170899** | ***SPOCK1*** | **136376050** | **0.004** |
| rs2262634 | *intergenic* | 134629551 | 0.004 |
| rs1345692 | *intergenic* | 128661180 | 0.004 |
| rs6872196 | *RP11-541P9.3* | 162582264 | 0.004 |
| rs1469052 | *SLC27A6* | 128194934 | 0.005 |
| rs248662 | *ITK* | 156659806 | 0.01 |
| rs193730 | *intergenic* | 141154583 | 0.01 |
| rs10057716 | *intergenic* | 128607170 | 0.01 |
| rs2115477 | *FSTL4* | 132837629 | 0.01 |
| rs12517292 | *ZCCHC10* | 132355147 | 0.01 |
| rs252095 | *RNF14* | 141362162 | 0.01 |
| RS10875553 | *TIGD6* | 149374932 | 0.01 |
| rs2351749 | *intergenic* | 153904423 | 0.01 |
| rs1030154 | *intergenic* | 165051330 | 0.01 |
| rs6595424 | *SNX24* | 122326950 | 0.01 |
| rs2270812 | *THG1L* | 157164962 | 0.01 |
| rs2268582 | *GABRG2* | 161515215 | 0.01 |
| rs888639 | *intergenic* | 163502963 | 0.01 |
| rs2530223 | *HDAC3* | 141014494 | 0.01 |
| rs13173675 | *GABRG2* | 161568643 | 0.01 |
| rs1560657 | *SYNPO* | 149991012 | 0.01 |
| rs10866717 | *FABP6* | 159633681 | 0.01 |
| rs1432693 | *intergenic* | 147609136 | 0.01 |
| **rs435571** | ***PPP2R2B*** | **146063448** | **0.01** |
| rs35524 | *MEGF10* | 126701622 | 0.01 |
| rs247456 | *intergenic* | 133374511 | 0.01 |
| rs17149748 | *SNX24* | 122267367 | 0.01 |
| rs193722 | *AC022120.1* | 160658223 | 0.01 |
| rs17166442 | *FSTL4* | 132581374 | 0.01 |
| rs30263 | *CHSY3* | 129495703 | 0.01 |
| rs1800449 | *SRFBP1* | 121413208 | 0.02 |
| rs17681747 | *FSTL4* | 132585528 | 0.02 |
| rs12186491 | *SPINK6* | 147593497 | 0.02 |
| rs6866231 | *ADAMTS19* | 128803345 | 0.02 |
| rs1006846 | *intergenic* | 133062239 | 0.02 |
| rs977960 | *intergenic* | 160709168 | 0.02 |
| rs4921307 | *ATP10B* | 160026777 | 0.02 |
| rs1560085 | *FSTL4* | 132566710 | 0.02 |
| **rs2120569** | ***PPP2R2B*** | **146073871** | **0.02** |
| rs40281 | *CTC-255N20.1* | 146586879 | 0.02 |
| rs1473132 | *intergenic* | 129669587 | 0.02 |
| rs25870 | *FSTL4* | 132784109 | 0.02 |
| rs17372823 | *intergenic* | 118158545 | 0.02 |
| rs1479585 | *DIAPH1* | 140976528 | 0.02 |
| rs2190578 | *CTB-1I21.2* | 136190484 | 0.02 |
| rs6871144 | *C1QTNF2* | 159790094 | 0.02 |
| rs1154826 | *intergenic* | 128689996 | 0.02 |
| rs919343 | *intergenic* | 120790102 | 0.02 |
| **rs17524553** | ***PPP2R2B*** | **146460691** | **0.02** |
| rs13155097 | *ABLIM3* | 148584486 | 0.02 |
| rs34019 | *AC005592.2* | 142000545 | 0.02 |
| rs962271 | *ATP10B* | 160054549 | 0.02 |
| rs17060096 | *GABRG2* | 161542609 | 0.02 |
| rs1990926 | *FABP6* | 159632751 | 0.02 |
| rs14359 | *PCDH1* | 141242633 | 0.02 |
| rs12653760 | *KLHL3* | 137032796 | 0.03 |
| rs34012 | *AC005592.2* | 141995777 | 0.03 |
| rs1862164 | *ADAMTS19* | 128813527 | 0.03 |
| rs17166682 | *FSTL4* | 132734942 | 0.03 |
| rs2034577 | *GALNT10* | 153766356 | 0.03 |
| rs970629 | *CTC-575N7.1* | 129118828 | 0.03 |
| rs2098610 | *intergenic* | 162058997 | 0.03 |
| rs254672 | *CLINT1* | 157246215 | 0.03 |
| rs110411 | *U7* | 129724371 | 0.03 |
| **rs4704963** | ***EBF1*** | **158247378** | **0.03** |
| rs185289 | *intergenic* | 159335852 | 0.03 |
| rs1551937 | *SNX24* | 122328421 | 0.03 |
| rs161557 | *CTB-57H20.1* | 143200053 | 0.03 |
| rs718410 | *FSTL4* | 132807672 | 0.03 |
| rs4958192 | *U6* | 133973889 | 0.03 |
| rs4705103 | *TCERG1* | 145890028 | 0.03 |
| rs11960775 | *MEGF10* | 126715206 | 0.03 |
| rs329168 | *intergenic* | 125133971 | 0.03 |
| rs6889792 | *RP11-510I6.2* | 120951488 | 0.03 |
| rs996036 | *intergenic* | 142083203 | 0.04 |
| rs959727 | *FSTL4* | 132566211 | 0.04 |
| rs405954 | *FSTL4* | 132942656 | 0.04 |
| rs42265 | *intergenic* | 128404226 | 0.04 |
| rs6872555 | *intergenic* | 135772456 | 0.04 |
| rs10793817 | *FSTL4* | 132838301 | 0.04 |
| rs828755 | *intergenic* | 162349848 | 0.04 |
| rs7723266 | *FAT2* | 150886725 | 0.04 |
| rs10077959 | *SRFBP1* | 121320272 | 0.04 |
| rs4958263 | *SEC24A* | 134007166 | 0.04 |
| rs917876 | *intergenic* | 141634276 | 0.04 |
| rs6580564 | *intergenic* | 148092126 | 0.05 |
| rs10078419 | *SRFBP1* | 121354221 | 0.05 |
| rs2190573 | *intergenic* | 136170977 | 0.05 |
| rs4576132 | *intergenic* | 158984239 | 0.05 |
| rs10056380 | *intergenic* | 149034018 | 0.05 |
| rs26825 | *intergenic* | 122616212 | 0.05 |
| rs479632 | *CTC-349C3.1* | 134364518 | 0.05 |
| rs2905589 | *KLHL3* | 137018387 | 0.05 |
| rs11242158 | *FSTL4* | 132835992 | 0.05 |
| rs17351902 | *SRFBP1* | 121360503 | 0.05 |
