# Supplementary material for: Fine mapping of a linkage peak with integration of lipid traits identifies novel coronary artery disease genes on chromosome 5
Source: BMC Genet. 2012 Feb 27;13:12. doi: 10.1186/1471-2156-13-12 (PMC3309961; doi:10.1186/1471-2156-13-12)
Supplement: Additional file 5 — Linkage results for SNPs with early-onset CAD in the GENECARD cohort. Displayed are linkage results for the GENECARD sample. All SNPs with two-point lod scores ≥ 1.5 are listed followed by their genic location, base pair position, individual lod score, and genetic model used. [file 1471-2156-13-12-S5.DOCX]

**Additional File 5**

**Table S1. Linkage results for SNPs with early-onset CAD in the GENECARD cohort.** Displayed are linkage results for the GENECARD sample. All SNPs with two-point lod scores ≥ 1.5 are listed followed by their genic location, base pair position, individual lod score, and genetic model used.

|  |  |  |  |  |
| --- | --- | --- | --- | --- |
| **SNP** | **Gene** | **Physical Location** | **LOD Score** | **Disease Model** |
| rs17166444 | *FSTL4* | 132581687 | 2.1 | Dominant |
| **rs7736046** | ***PRELID2*** | **144971610** | **2.1** | **Recessive** |
| rs17166460 | *FSTL4* | 132588868 | 2.0 | Dominant |
| rs4958109 | *FSTL4* | 132573216 | 2.0 | Recessive |
| rs4958112 | *FSTL4* | 132609517 | 1.9 | Recessive |
| rs13718 | *HSPA4* | 132441302 | 1.8 | Recessive |
| **rs1919515** | ***SPOCK1*** | **136822835** | **1.8** | **Dominant** |
| rs12189066 | *CHSY3* | 129293427 | 1.8 | Dominant |
| rs6596097 | *HSPA4* | 132392393 | 1.8 | Dominant |
| rs12515798 | *PCBD2* | 134305197 | 1.7 | Recessive |
| **rs6866539** | ***PRELID2*** | **144943205** | **1.7** | **Recessive** |
| rs330683 | *intergenic* | 123208563 | 1.6 | Recessive |
| rs9687567 | *GRAMD3* | 125800646 | 1.6 | Recessive |
| rs4705986 | *ZCCHC10* | 132349654 | 1.6 | Dominant |
| rs25870 | *FSTL4* | 132784109 | 1.6 | Recessive |
| rs6596102 | *HSPA4* | 132415569 | 1.5 | Dominant |
