# Supplementary material for: Fine mapping of a linkage peak with integration of lipid traits identifies novel coronary artery disease genes on chromosome 5
Source: BMC Genet. 2012 Feb 27;13:12. doi: 10.1186/1471-2156-13-12 (PMC3309961; doi:10.1186/1471-2156-13-12)
Supplement: Additional file 6 — Association of SNPs with CAD in the CATHGEN Cohort. Displayed are all significant results for the case-control analysis of SNPs with CAD in the CATHGEN sample. Each significant SNP is listed followed by their genic location, base pair, p-value, odds ratio, and 95% confidence interval for the genotypic and allelic models, respectively. [file 1471-2156-13-12-S6.DOCX]

**Additional File 6**

**Table S1. Association of SNPs with CAD in the CATHGEN Cohort.** Displayed are all significant results for the case-control analysis of SNPs with CAD in the CATHGEN sample. Each significant SNP is listed followed by their genic location, base pair, p-value, odds ratio, and 95% confidence interval for the genotypic and allelic models, respectively.

|  |  |  | **Genotypic** | | **Allelic** | |
| --- | --- | --- | --- | --- | --- | --- |
| **SNP** | **Gene** | **Physical Location** | **P-value** | **OR (CI)** | **P-value** | **OR (CI)** |
| rs10050603 | *intergenic* | 129531748 | 0.0002 | 0.56 (0.42-0.76) | 0.0002 | 0.54 (0.38-0.75) |
| rs1004965 | *FBN2* | 127814937 | 0.001 | 0.70 (0.57-0.86) | 0.02 | 0.72 (0.54-0.95) |
| rs10073062 | *FBN2* | 127859574 | 0.001 | 0.70 (0.57-0.85) | 0.04 | 0.74 (0.56-.98) |
| rs13354399 | *GRIA1* | 152989878 | 0.002 | 1.63 (1.19-2.24) | 0.001 | 1.79 (1.27-2.52) |
| rs1059110 | *CTNNA1* | 138159344 | 0.002 | 0.74 (0.61-0.89) | 0.03 | 0.75 (0.58-0.974) |
| rs10059749 | *intergenic* | 138063241 | 0.003 | 0.74 (0.61-0.90) | 0.02 | 0.74 (0.57-0.958) |
| rs13173675 | *GABRG2* | 161582661 | 0.003 | 1.34 (1.10-1.62) | 0.02 | 1.38 (1.05-1.8) |
| **rs13179436** | ***PRELID2*** | **144924504** | **0.003** | **1.45 (1.13-1.86)** | **0.005** | **1.52 (1.13-2.032)** |
| rs1006846 | *intergenic* | 125845364 | 0.004 | 1.32 (1.09-1.59) | 0.01 | 1.45 (1.11-1.893) |
| rs1972072 | *CTNNA1* | 138123994 | 0.004 | 0.75 (0.62-0.91) | 0.03 | 0.75 (0.57-0.965) |
| rs1034664 | *KLHL3* | 137065324 | 0.004 | 0.73 (0.6--907) | 0.02 | 0.73 (0.56-0.941) |
| rs26159 | *CTC-806A22.1* | 144763704 | 0.004 | 0.75 (0.63-.915) | 0.03 | 0.74 (0.57-0.967) |
| **rs10302810** | ***PPP2R2B*** | **146169511** | **0.004** | **0.75 (0.62-0.91)** | **0.04** | **0.74 (0.55-0.985)** |
| rs10073634 | *intergenic* | 134599977 | 0.004 | 0.74 (0.61-0.91) | 0.08 | 0.78 (0.59-1.031) |
| **rs1541659** | ***PRELID2*** | **144883076** | **0.005** | **1.40 (1.11-1.78)** | **0.01** | **1.45 (1.09-1.913)** |
| rs11949017 | *FBN2* | 127761510 | 0.005 | 0.71 (0.57-0.90) | 0.004 | 0.67 (0.51-0.88) |
| rs10078499 | *intergenic* | 121104400 | 0.01 | 1.55 (1.14-2.11) | 0.001 | 1.83 (1.27-2.638) |
| rs251021 | *DIAPH1* | 140899268 | 0.01 | 0.62 (0.44-0.86) | 0.01 | 0.62 (0.43-0.881) |
| rs10039139 | *RP11-381K20.2* | 137164863 | 0.01 | 0.73 (0.59-0.91) | 0.02 | 0.72 (0.55-0.943) |
| rs423469 | *CTNNA1* | 138185293 | 0.01 | 0.76 (0.63-.925) | 0.04 | 0.76 (0.59-0.985) |
| rs6877187 | *CTNNA1* | 138103266 | 0.01 | 0.76 (0.63-0.92) | 0.04 | 0.77 (0.59-0.988) |
| rs11743060 | *SGCD* | 155784910 | 0.01 | 1.31 (1.07-1.58) | 0.01 | 1.45 (1.09-1.911) |
| rs700629 | *CTNNA1* | 138133869 | 0.01 | 0.76 (0.63-0.92) | 0.05 | 0.78 (0.60-0.999) |
| rs11167643 | *intergenic* | 146921478 | 0.01 | 0.72 (0.57-0.91) | 0.02 | 0.73 (0.55-0.953) |
| rs11242397 | *intergenic* | 134998909 | 0.01 | 1.33 (1.08-1.64) | 0.002 | 1.52 (1.16-1.978) |
| rs10064637 | *AC005592.2* | 141910036 | 0.01 | 0.75 (0.61-0.92) | 0.01 | 0.71 (0.54-0.922) |
| **rs443033** | ***PRELID2*** | **144936522** | **0.01** | **1.29 (1.07-1.56)** | **0.02** | **1.37 (1.05-1.787)** |
| rs11575949 | *PCDHGA12* | 140795128 | 0.01 | 0.60 (0.42-0.87) | 0.02 | 0.64 (0.44-.937) |
| rs1277 | *SLC22A4* | 131670546 | 0.01 | 0.77 (0.64-0.93) | 0.1 | 0.8 (0.61-.041) |
| rs10479044 | *FSTL4* | 132895893 | 0.01 | 0.77 (0.63-0.93) | 0.08 | 0.79 (0.60-1.028) |
| rs825751 | *CTNNA1* | 138266546 | 0.01 | 0.77 (0.64-0.93) | 0.05 | 0.78 (0.60-1.002) |
| rs6861341 | *SIL1* | 138291405 | 0.01 | 0.78 (0.65-0.94) | 0.13 | 0.81 (0.62-1.061) |
| rs11954297 | *RBM27* | 145594215 | 0.01 | 1.28 (1.06-1.55) | 0.003 | 1.47 (1.13-1.9) |
| rs1154826 | *intergenic* | 137107734 | 0.01 | 0.75 (0.60-0.93) | 0.01 | 0.72 (0.55-0.927) |
| rs11749892 | *intergenic* | 128689996 | 0.01 | 1.59 (1.12-2.27) | 0.01 | 1.7 (1.15-2.486) |
| **rs11167956** | ***PPP2R2B*** | **146185874** | **0.01** | **0.69 (0.53-0.91)** | **0.01** | **0.67 (0.49-0.914)** |
| rs1042665 | *HSPA9* | 137902339 | 0.01 | 1.32 (1.06-1.63) | 0.01 | 1.45 (1.11-1.876) |
| **rs1383169** | ***PPP2R2B*** | **146073871** | **0.01** | **0.77 (0.64-.944)** | **0.02** | **0.71 (0.52-0.956)** |
| rs851278 | *CTNNA1* | 138234116 | 0.01 | 0.78 (0.64-0.94) | 0.09 | 0.8 (0.61-1.033) |
| **rs10447210** | ***EBF1*** | **158502728** | **0.01** | **1.26 (1.05-1.52)** | **0.01** | **1.49 (1.11-1.994)** |
| rs1181962 | *intergenic* | 157879264 | 0.01 | 0.79 (0.65-0.94) | 0.05 | 0.76 (0.57-1) |
| rs6595174 | *DMXL1* | 118532034 | 0.01 | 1.28 (1.05-1.55) | 0.02 | 1.35 (1.04-1.751) |
| rs257906 | *SLC27A6* | 128337023 | 0.01 | 0.74 (0.59-0.93) | 0.02 | 0.73 (0.55-0.949) |
| rs12188371 | *intergenic* | 138020898 | 0.01 | 0.77 (0.63-.946) | 0.05 | 0.77 (0.59-.997) |
| rs6595178 | *DMXL1* | 118440675 | 0.01 | 1.26 (1.05-.522) | 0.01 | 1.46 (1.10-1.939) |
| rs17114681 | *GRIA1* | 152960528 | 0.01 | 0.68 (0.50-0.92) | 0.01 | 0.64 (0.46-0.891) |
| rs7717375 | *SIL1* | 138482506 | 0.01 | 1.25 (1.04-1.49) | 0.06 | 1.31 (0.99-1.73) |
| rs1431945 | *intergenic* | 154547570 | 0.01 | 0.77 (0.63-.948) | 0.03 | 0.75 (0.58-0.972) |
| rs17096590 | *RBM27* | 145643048 | 0.01 | 1.26 (1.04-1.52) | 0.005 | 1.45 (1.11-1.871) |
| rs13183976 | *RBM27* | 145693202 | 0.01 | 1.26 (1.04-1.52) | 0.01 | 1.42 (1.09-1.836) |
| **rs2107443** | ***SPOCK1*** | **136319856** | **0.01** | **0.79 (0.66-.955)** | **0.03** | **0.73 (0.55-.965)** |
| rs1564192 | *intergenic* | 137991852 | 0.01 | 0.77 (0.63-0.95) | 0.05 | 0.77 (0.59-0.995) |
| rs1432631 | *intergenic* | 128386801 | 0.01 | 1.30 (1.05-1.61) | 0.24 | 1.18 (0.89-1.54) |
| rs272879 | *SLC22A4* | 131663062 | 0.02 | 0.79 (0.65-0.95) | 0.07 | 0.78 (0.59-1.019) |
| rs160399 | *intergenic* | 155458803 | 0.02 | 0.76 (0.60-0.95) | 0.02 | 0.72 (0.55-0.94) |
| rs768232 | *TCERG1* | 145875259 | 0.02 | 0.66 (0.47-0.93) | 0.01 | 0.64 (0.44-0.911) |
| rs10515456 | *AC034220.3* | 131699669 | 0.02 | 1.56 (1.07-2.25) | 0.02 | 1.58 (1.07-2.315) |
| rs7875 | *HNRNPA0* | 137088049 | 0.02 | 0.76 (0.61-0.95) | 0.02 | 0.74 (0.56-0.959) |
| rs17099156 | *AC005592.2* | 142043752 | 0.02 | 1.29 (1.04-1.6) | 0.18 | 1.2 (0.92-1.567) |
| rs258768 | *ARHGAP26* | 142561294 | 0.02 | 0.77 (0.62-0.95) | 0.04 | 0.76 (0.58-.988) |
| **rs1544754** | ***EBF1*** | **158414305** | **0.02** | **1.31 (1.04-1.66)** | **0.04** | **1.34 (1.01-1.76)** |
| rs17118082 | *intergenic* | 139701920 | 0.02 | 0.80 (0.67-0.96) | 0.18 | 0.82 (0.61-1.096) |
| rs11334 | *KDM3B* | 137742465 | 0.02 | 1.27 (1.04-.57) | 0.02 | 1.39 (1.06-1.815) |
| rs17537018 | *intergenic* | 125133971 | 0.02 | 0.80 (0.67-0.96) | 0.24 | 0.86 (0.65-1.113) |
| rs249732 | *AC005592.2* | 142022777 | 0.02 | 0.74 (0.57-0.95) | 0.07 | 0.77 (0.58-1.025) |
| rs7444042 | *CTC-806A22.1* | 144738094 | 0.02 | 1.26 (1.03-1.53) | 0.01 | 1.42 (1.1--835) |
| rs11242158 | *FSTL4* | 132706629 | 0.02 | 1.30 (1.04-1.64) | 0.02 | 1.38 (1.04-1.81) |
| rs1859067 | *intergenic* | 135009714 | 0.02 | 1.30 (1.04-.645) | 0.01 | 1.43 (1.09-.877) |
| rs10050399 | *GRAMD3* | 125826191 | 0.02 | 1.28 (1.03-1.60) | 0.01 | 1.44 (1.10-1.879) |
| **rs17635991** | ***EBF1*** | **158477678** | **0.02** | **0.73 (0.56-.958)** | **0.03** | **0.72 (0.53-0.973)** |
| rs211037 | *GABRG2* | 161528280 | 0.03 | 1.27 (1.03-.565) | 0.01 | 1.41 (1.08-1.829) |
| rs30708 | *ADAMTS19* | 128968594 | 0.03 | 1.68 (1.06-2.64) | 0.04 | 1.65 (1.03-2.634) |
| rs2081967 | *intergenic* | 159036298 | 0.03 | 0.80 (0.66-0.97) | 0.07 | 0.78 (0.59-1.02) |
| rs274546 | *AC034220.3* | 131699867 | 0.03 | 0.80 (0.67-0.97) | 0.16 | 0.83 (0.63-1.076) |
| rs12652669 | *SGCD* | 155862225 | 0.03 | 1.32 (1.03-1.68) | 0.04 | 1.35 (1.01-1.805) |
| rs12514133 | *KDM3B* | 137754695 | 0.03 | 0.81 (0.68-.975) | 0.05 | 0.77 (0.58-1.005) |
| **rs31862** | ***EBF1*** | **158345923** | **0.03** | **1.30 (1.03-1.65)** | **0.02** | **1.39 (1.04-1.844)** |
| rs245581 | *intergenic* | 141577451 | 0.03 | 1.57 (1.05-2.34) | 0.03 | 1.59 (1.04-2.426) |
| rs249660 | *intergenic* | 153222799 | 0.03 | 0.80 (0.66-0.97) | 0.08 | 0.79 (0.61-1.026) |
| rs1428143 | *FSTL4* | 132843679 | 0.03 | 0.68 (0.48-0.96) | 0.03 | 0.67 (0.47-0.963) |
| rs10213948 | *GRAMD3* | 125760722 | 0.03 | 0.76 (0.59-0.97) | 0.06 | 0.77 (0.58-.015) |
| rs4835678 | *KDM3B* | 137772296 | 0.03 | 1.25 (1.02-1.54) | 0.02 | 1.36 (1.04-.769) |
| rs283424 | *intergenic* | 154894913 | 0.03 | 1.28 (1.02-1.60) | 0.08 | 1.27 (0.97-1.646) |
| rs11575963 | *PCDHGA12* | 140735215 | 0.03 | 1.38 (1.03-1.84) | 0.05 | 1.4 (1-1.-6) |
| RS164080 | *GNPDA1* | 141391532 | 0.03 | 0.81 (0.68-0.98) | 0.45 | 0.89 (0.66-1.197) |
| rs4836434 | *SLC27A6* | 128362037 | 0.03 | 0.79 (0.63-0.97) | 0.04 | 0.76 (0.58-0.988) |
| rs1858079 | *FBN2* | 127802836 | 0.03 | 0.80 (0.66-0.98) | 0.25 | 0.84 (0.61-1.138) |
| rs249749 | *AC005592.2* | 142019596 | 0.03 | 0.79 (0.64-0.98) | 0.11 | 0.8 (0.61-1.048) |
| rs401750 | *GABRG2* | 161580983 | 0.03 | 1.25 (1.02-.555) | 0.15 | 1.22 (0.93-1.594) |
| rs6865969 | ***EBF1*** | 158418490 | 0.03 | 1.23 (1.01-1.49) | 0.04 | 1.32 (1.00-1.734) |
| rs29550 | *FSTL4* | 132616959 | 0.03 | 0.81 (0.67-0.98) | 0.04 | 0.75 (0.56-0.99) |
| rs924581 | *HAND1* | 153855889 | 0.03 | 0.73 (0.54-0.97) | 0.13 | 0.78 (0.57-.071) |
| **rs2189597** | ***SPOCK1*** | **136422213** | **0.03** | **0.81 (0.67-0.98)** | **0.04** | **0.75 (0.57-0.98)** |
| rs17475118 | *CTB-73N10.1* | 144807495 | 0.03 | 0.8 (0.65-.983) | 0.1 | 0.8 (0.61-1.046) |
| **rs7713855** | ***PRELID2*** | **144971610** | **0.03** | **1.23 (1.01-1.50)** | **0.04** | **1.34 (1.01-1.769)** |
| rs10117 | *AC011385.3* | 137892170 | 0.03 | 1.22 (1.01-1.47) | 0.09 | 1.27 (0.96-1.686) |
| **rs6872714** | ***SPOCK1*** | **136425966** | **0.03** | **1.23 (1.01-1.50)** | **0.36** | **1.14 (0.86-1.503)** |
| rs6580060 | *GALNT10* | 153596453 | 0.04 | 1.21 (1.01-1.46) | 0.07 | 1.29 (0.98-.709) |
| rs2901316 | *intergenic* | 138036255 | 0.04 | 0.79 (0.63-0.98) | 0.05 | 0.77 (0.59-1.003) |
| rs7447475 | *SGCD* | 155835122 | 0.04 | 1.30 (1.01-1.66) | 0.05 | 1.32 (0.99-1.747) |
| **rs6869051** | ***EBF1*** | **158222866** | **0.04** | **0.72 (0.52-0.98)** | **0.03** | **0.69 (0.48-0.964)** |
| rs7726234 | *KDM3B* | 137708434 | 0.04 | 0.82 (0.69-.988) | 0.09 | 0.79 (0.60-1.037) |
| rs154076 | *ETF1* | 137863134 | 0.04 | 1.22 (1.01-1.47) | 0.1 | 1.27 (0.95-1.676) |
| **rs160974** | ***PPP2R2B*** | **146434800** | **0.04** | **0.76 (0.59-0.98)** | **0.01** | **0.67 (0.49-0.907)** |
| rs2914621 | *intergenic* | 148257612 | 0.04 | 1.36 (1.01-1.81) | 0.05 | 1.39 (1.00-1.92) |
| rs274554 | *SLC22A5* | 131724950 | 0.04 | 1.27 (1.01-1.60) | 0.06 | 1.32 (0.99-1.746) |
| rs30263 | *CHSY3* | 129495703 | 0.04 | 0.76 (0.59-0.98) | 0.04 | 0.74 (0.55-0.993) |
| rs233031 | *PRRC1* | 126887569 | 0.04 | 1.23 (1.01-1.51) | 0.01 | 1.4 (1.08-1.805) |
| rs2905589 | *KLHL3* | 136987023 | 0.04 | 1.22 (1.01-.482) | 0.04 | 1.31 (1.01-1.699) |
| rs30270 | *intergenic* | 144782407 | 0.04 | 0.82 (0.68-0.99) | 0.11 | 0.8 (0.60-1.05) |
| rs27456 | *FBN2* | 127809413 | 0.04 | 1.35 (1.01-1.79) | 0.04 | 1.38 (1.00-1.879) |
| rs4958192 | *U6* | 133973889 | 0.04 | 0.80 (0.65-0.99) | 0.1 | 0.8 (0.60-1.042) |
| rs7706091 | *SGCD* | 155876204 | 0.04 | 0.68 (0.48-.985) | 0.01 | 0.61 (0.41-0.904) |
| rs4476720 | *GRAMD3* | 125810777 | 0.04 | 0.80 (0.65-0.99) | 0.19 | 0.84 (0.64-1.09) |
| rs433759 | *FSTL4* | 132835992 | 0.04 | 1.26 (1.00-1.58) | 0.06 | 1.29 (0.98-1.699) |
| rs4705986 | *ZCCHC10* | 132349654 | 0.04 | 1.66 (1.01-2.73) | 0.05 | 1.66 (1.00-2.735) |
| rs418210 | *GABRG2* | 161568643 | 0.04 | 0.76 (0.58-0.99) | 0.05 | 0.74 (0.54-0.997) |
| rs2304029 | *FAT2* | 150891772 | 0.04 | 0.66 (0.45-.987) | 0.03 | 0.65 (0.43-0.959) |
| rs2546968 | *AC008706.1* | 159552789 | 0.04 | 1.20 (1.00-1.44) | 0.52 | 1.09 (0.83-1.427) |
| rs17057519 | *FABP6* | 159637815 | 0.04 | 0.68 (0.46-0.99) | 0.08 | 0.71 (0.48-1.048) |
| rs3777378 | *KLHL3* | 136971256 | 0.04 | 0.78 (0.62-0.99) | 0.05 | 0.76 (0.57-0.998) |
| rs329168 | *intergenic* | 153939449 | 0.05 | 2.07 (1.01-4.22) | 0.05 | 2.07 (1.01-4.228) |
| rs10476976 | *AC034205.1* | 150747877 | 0.05 | 0.81 (0.65-0.99) | 0.22 | 0.84 (0.63-1.108) |
| rs7731883 | *SGCD* | 155788816 | 0.05 | 0.82 (0.69-.997) | 0.28 | 0.87 (0.66-1.128) |
| rs12659166 | *PPARGC1B* | 149106944 | 0.05 | 1.44 (1.00-2.07) | 0.08 | 1.4 (0.96-2.03) |
| rs2545025 | *HDAC3* | 141011346 | 0.05 | 0.74 (0.55-.996) | 0.05 | 0.73 (0.52-0.998) |
| rs219287 | *ETF1* | 137837245 | 0.05 | 1.20 (1.00-1.44) | 0.003 | 1.49 (1.14-1.947) |
| rs7729098 | *RBM27* | 145668298 | 0.05 | 1.24 (1.00-1.55) | 0.07 | 1.29 (0.98-1.684) |
| rs42265 | *intergenic* | 137818178 | 0.05 | 1.2 (1.00-1.43) | 0.01 | 1.45 (1.09-1.902) |
| rs652371 | *intergenic* | 133160254 | 0.05 | 1.21 (1.00-1.46) | 0.2 | 1.19 (0.91-1.546) |
| rs7721110 | *SIL1* | 138436816 | 0.05 | 0.73 (0.54-0.99) | 0.05 | 0.72 (0.52-) |
| rs4836492 | *CHSY3* | 129460664 | 0.05 | 0.83 (0.69-0.99) | 0.19 | 0.82 (0.61-1.1) |
| rs6556349 | *intergenic* | 154981996 | 0.05 | 1.21 (1.00-1.46) | 0.01 | 1.41 (1.07-1.853) |
| rs6873053 | *TIMD4* | 156376703 | 0.05 | 1.48 (1.00-2.20) | 0.1 | 1.42 (0.93-2.147) |
| rs3776070 | *SLC26A2* | 149361221 | 0.05 | 0.79 (0.63-1.00) | 0.05 | 0.76 (0.58-.999) |
| **rs161042** | ***PPP2R2B*** | **146413413** | **0.06** | **0.81 (0.65-1.00)** | **0.05** | **0.77 (0.59-0.999)** |
| rs998051 | *TCERG1* | 145823141 | 0.06 | 0.77 (0.59-1.00) | 0.05 | 0.74 (0.54-0.995) |
| rs6893408 | *AC005592.2* | 141887551 | 0.06 | 0.82 (0.67-1.00) | 0.03 | 0.75 (0.57-0.972) |
| rs10900864 | *DNAJC18* | 138773090 | 0.06 | 1.19 (0.99-1.44) | 0.01 | 1.45 (1.10-1.901) |
| rs6580470 | *intergenic* | 121151193 | 0.06 | 1.35 (0.98-1.85) | 0.01 | 1.7 (1.12-2.577) |
| rs7522 | *PRRC1* | 126865295 | 0.06 | 1.20 (0.99-.478) | 0.01 | 1.38 (1.07-1.78) |
| rs17171718 | *FAM13B* | 137373502 | 0.06 | 1.32 (0.98-1.79) | 0.05 | 1.4 (1.00-1.942) |
| rs349691 | *KCTD16* | 143845254 | 0.06 | 0.84 (0.7--01) | 0.05 | 0.76 (0.57-0.995) |
| rs7701443 | *NR3C1* | 142792650 | 0.06 | 1.19 (0.98-1.43) | 0.02 | 1.37 (1.04-1.791) |
| rs272893 | *SLC22A4* | 131671662 | 0.07 | 1.65 (0.96--2.8) | 0.05 | 1.77 (100.--3.10) |
| rs6595754 | *intergenic* | 152847022 | 0.07 | 1.22 (0.98-1.53) | 0.04 | 1.32 (1.00-1.73) |
| rs6887040 | *intergenic* | 121903853 | 0.07 | 0.80 (0.63-1.01) | 0.03 | 0.75 (0.57-.977) |
| rs6890204 | *intergenic* | 157493461 | 0.07 | 1.34 (0.97-1.86) | 0.04 | 1.45 (1.01-2.09) |
| rs6863481 | *CHSY3* | 129364818 | 0.07 | 0.84 (0.70-1.01) | 0.05 | 0.75 (0.56-0.998) |
| rs4526101 | *GRAMD3* | 125818698 | 0.08 | 1.18 (0.98-1.43) | 0.02 | 1.39 (1.06-1.809) |
| **rs985267** | ***SPOCK1*** | **136387259** | **0.08** | **0.83 (0.67-1.02)** | **0.03** | **0.73 (0.55-0.97)** |
| rs735620 | *intergenic* | 133062239 | 0.08 | 1.19 (0.97-1.46) | 0.03 | 1.32 (1.02-.706) |
| **rs7736443** | ***EBF1*** | **158222797** | **0.08** | **1.23 (0.97-1.55)** | **0.05** | **1.33 (1.00-1.752)** |
| rs739954 | *intergenic* | 153912076 | 0.08 | 1.17 (0.97-1.42) | 0.01 | 1.42 (1.09-1.842) |
| rs432792 | *Y_RNA* | 127703136 | 0.09 | 0.81 (0.63-1.03) | 0.03 | 0.74 (0.55-0.977) |
| rs744675 | *intergenic* | 126596610 | 0.09 | 1.16 (0.97-1.40) | 0.003 | 1.53 (1.15-2.023) |
| rs741146 | *SH3TC2* | 148307111 | 0.1 | 1.17 (0.97-1.41) | 0.04 | 1.31 (1.01-.688) |
| rs17114874 | *GRIA1* | 153189431 | 0.1 | 1.20 (0.96-1.51) | 0.03 | 1.34 (1.02-1.747) |
| rs587410 | *ADAMTS19* | 128960082 | 0.11 | 0.84 (0.68-1.04) | 0.02 | 0.72 (0.55-0.942) |
| rs4365836 | *AC135457.1* | 138732103 | 0.12 | 1.16 (0.96-1.39) | 0.01 | 1.39 (1.06-1.818) |
| rs7709766 | *KLHL3* | 137018387 | 0.13 | 0.86 (0.72-1.04) | 0.01 | 0.7 (0.52-0.927) |
| rs905788 | *SGCD* | 155779168 | 0.13 | 0.86 (0.71-1.04) | 0.05 | 0.74 (0.55-0.994) |
| rs17518831 | *GRIA1* | 152887567 | 0.13 | 0.86 (0.71-1.04) | 0.01 | 0.68 (0.52-0.892) |
| rs7703330 | *intergenic* | 128404226 | 0.14 | 0.87 (0.72-1.04) | 0.02 | 0.7 (0.52-.939) |
| rs4835728 | *DNAJC18* | 138754741 | 0.14 | 1.15 (0.95-1.38) | 0.02 | 1.38 (1.05-1.795) |
| rs7710890 | *intergenic* | 129892696 | 0.16 | 0.87 (0.72-1.05) | 0.02 | 0.7 (0.52-0.945) |
| rs3805653 | *FBN2* | 127635867 | 0.16 | 0.87 (0.72-.055) | 0.05 | 0.73 (0.53-0.998) |
| **rs7736046** | ***PRELID2*** | **145210041** | **0.18** | **0.86 (0.70-1.07)** | **0.05** | **0.76 (0.58-0.997)** |
| rs11241774 | *RP11-284A20.2* | 124207748 | 0.18 | 0.88 (0.73-1.06) | 0.05 | 0.76 (0.57-0.999) |
| **rs2120569** | ***PPP2R2B*** | **146118907** | **0.19** | **0.87 (0.72-.066)** | **0.02** | **0.72 (0.55-.945)** |
| rs4836257 | *GRAMD3* | 125799833 | 0.19 | 0.87 (0.72-1.06) | 0.04 | 0.75 (0.57-0.981) |
| rs7714592 | *intergenic* | 118058065 | 0.2 | 1.12 (0.94-1.34) | 0.04 | 1.33 (1.01-1.753) |
| rs813188 | *intergenic* | 157114139 | 0.2 | 1.12 (0.94-.351) | 0.01 | 1.46 (1.10-1.92) |
| rs42395 | *LEAP2* | 132214330 | 0.21 | 1.13 (0.93-1.36) | 0.02 | 1.44 (1.06-1.94) |
| rs977776 | *intergenic* | 126578538 | 0.23 | 1.11 (0.93-1.33) | 0.03 | 1.38 (1.03-1.838) |
| rs17519656 | *GRIA1* | 152951592 | 0.24 | 1.12 (0.92-1.35) | 0.04 | 1.36 (1.01-1.823) |
| rs984833 | *intergenic* | 117764534 | 0.29 | 1.10 (0.92-.32) | 0.03 | 1.39 (1.04-1.847) |
| rs33409 | *SYNPO* | 150038265 | 0.34 | 0.91 (0.77-1.09) | 0.04 | 0.73 (0.55-0.979) |
