# Supplementary material for: Fine mapping of a linkage peak with integration of lipid traits identifies novel coronary artery disease genes on chromosome 5
Source: BMC Genet. 2012 Feb 27;13:12. doi: 10.1186/1471-2156-13-12 (PMC3309961; doi:10.1186/1471-2156-13-12)
Supplement: Additional file 7 — Association of SNPs with CAD for GENECARD proband vs. CATHGEN controls sample set. Displayed are the results of the GENECARD proband-CATHGEN control analysis, with all significant SNPs listed followed by their genic location, base pair position, p-value, odds ratio, and 95% confidence intervals for the genotypic and allelic models used. [file 1471-2156-13-12-S7.DOCX]

**Additional File 7**

**Table S1. Association of SNPs with CAD for GENECARD proband vs. CATHGEN controls sample set.** Displayed are the results of the GENECARD proband-CATHGEN control analysis, with all significant SNPs listed followed by their genic location, base pair position, p-value, odds ratio, and 95% confidence intervals for the genotypic and allelic models used.

|  |  |  | **Genotypic** | | **Allelic** | |
| --- | --- | --- | --- | --- | --- | --- |
| **SNP** | **Gene** | **Physical Location** | **P-value** | **OR(CI)** | **P-value** | **OR(CI)** |
| rs6895094 | *ARAP3* | 141037277 | 0.0003 | 0.58 (0.43-0.78) | 0.0001 | 0.44 (0.29-0.67) |
| rs11744339 | *CTB-99A3.1* | 145978081 | 0.0003 | 2.31 (1.47-3.64) | 0.001 | 2.27 (1.41-3.66) |
| rs3763120 | *ARAP3* | 141049407 | 0.001 | 0.59 (0.43-0.80) | 0.001 | 0.48 (0.31-0.75) |
| rs17537018 | *intergenic* | 155458803 | 0.001 | 0.52 (0.35-0.78) | 0.002 | 0.51 (0.33-0.78) |
| rs11738917 | *PPARGC1B* | 149155039 | 0.002 | 2.35 (1.37-4.01) | 0.003 | 2.39 (1.35-4.22) |
| rs1479585 | *DIAPH1* | 140976528 | 0.002 | 1.56 (1.17-2.08) | 0.01 | 1.71 (1.11-2.62) |
| rs2530223 | *HDAC3* | 141014494 | 0.003 | 1.55 (1.16-2.06) | 0.01 | 1.74 (1.13-2.67) |
| rs10515456 | *AC034220.3* | 131699669 | 0.003 | 2.12 (1.28-3.51) | 0.003 | 2.19 (1.30-3.70) |
| rs4426908 | *AC004237.1* | 132022718 | 0.003 | 0.6 (0.42-0.84) | 0.01 | 0.59 (0.39-0.89) |
| rs7737127 | *GRAMD3* | 125764139 | 0.004 | 0.55 (0.36-0.82) | 0.01 | 0.52 (0.32-0.84) |
| rs6864396 | *AC004237.1* | 132047875 | 0.005 | 0.60 (0.43-0.85) | 0.01 | 0.56 (0.37-0.85) |
| rs32929 | *intergenic* | 141107382 | 0.005 | 1.61 (1.16-2.24) | 0.01 | 1.77 (1.15-2.72) |
| rs17208502 | *ARAP3* | 141043299 | 0.005 | 0.34 (0.16-0.72) | 0.01 | 0.33 (0.15-0.72) |
| rs11743259 | *CDC25C* | 137636706 | 0.005 | 1.68 (1.17-2.41) | 0.02 | 1.63 (1.06-2.49) |
| rs9313882 | *GABRB2* | 160870473 | 0.01 | 0.58 (0.40-0.86) | 0.02 | 0.59 (0.38-0.91) |
| rs184946 | *intergenic* | 158079394 | 0.01 | 1.58 (1.13-2.20) | 0.01 | 1.79 (1.15-2.80) |
| **rs17712788** | ***EBF1*** | **158182330** | **0.01** | **1.67 (1.15-2.42)** | **0.04** | **1.59 (1.02-2.45)** |
| rs2910291 | *GABRB2* | 160812482 | 0.01 | 0.59 (0.40-0.86) | 0.01 | 0.56 (0.36-0.86) |
| rs248647 | *intergenic* | 141105691 | 0.01 | 0.68 (0.51-0.90) | 0.01 | 0.56 (0.37-0.86) |
| rs10039139 | *RP11-381K20.2* | 137164863 | 0.01 | 0.61 (0.42-0.88) | 0.01 | 0.54 (0.35-0.84) |
| rs621583 | *intergenic* | 134597660 | 0.01 | 1.45 (1.09-1.92) | 0.07 | 1.49 (0.96-2.32) |
| rs11747454 | *SIL1* | 138318158 | 0.01 | 0.59 (0.40-0.88) | 0.004 | 0.50 (0.32-0.80) |
| rs11241891 | *GRAMD3* | 125768020 | 0.01 | 0.62 (0.43-0.89) | 0.03 | 0.62 (0.40-0.95) |
| rs17519656 | *GRIA1* | 152960528 | 0.01 | 0.49 (0.28-0.85) | 0.02 | 0.50 (0.28-0.88) |
| rs10793817 | *FSTL4* | 132838301 | 0.01 | 1.46 (1.09-1.96) | 0.27 | 1.31 (0.80-2.15) |
| rs4705093 | *PPARGC1B* | 149179961 | 0.01 | 1.75 (1.13-2.72) | 0.03 | 1.68 (1.04-2.74) |
| **rs1383169** | ***PPP2R2B*** | **146434800** | **0.01** | **0.50 (0.30-0.86)** | **0.02** | **0.52 (0.30-0.90)** |
| rs2072315 | *PCDHGA12* | 140763029 | 0.01 | 1.45 (1.08-1.95) | 0.16 | 1.46 (0.85-2.48) |
| rs31340 | *FSTL4* | 132799163 | 0.01 | 0.21 (0.06-0.72) | 0.01 | 0.20 (0.06-0.71) |
| rs2240697 | *PCDHGA12* | 140751128 | 0.01 | 1.45 (1.08-1.94) | 0.26 | 1.34 (0.80-2.27) |
| **rs6884385** | ***SPOCK1*** | **136853589** | **0.01** | **1.48 (1.08-2.02)** | **0.01** | **1.65 (1.10-2.47)** |
| rs6650971 | *FAT2* | 150901261 | 0.02 | 1.44 (1.07-1.95) | 0.01 | 1.68 (1.12-2.53) |
| rs6595440 | *CEP120* | 122718736 | 0.02 | 0.70 (0.52-0.93) | 0.14 | 0.72 (0.47-1.10) |
| rs6580060 | *GALNT10* | 153596453 | 0.02 | 1.42 (1.06-1.91) | 0.06 | 1.52 (0.98-2.33) |
| rs2447820 | *SNX24* | 122264736 | 0.02 | 1.64 (1.09-2.48) | 0.03 | 1.67 (1.04-2.68) |
| rs4912870 | *AC005592.2* | 142015715 | 0.02 | 0.65 (0.46-0.92) | 0.06 | 0.66 (0.42-1.02) |
| rs6595424 | *SNX24* | 122326950 | 0.02 | 1.66 (1.09-2.51) | 0.03 | 1.67 (1.05-2.66) |
| rs10036623 | *GRIA1* | 152919943 | 0.02 | 1.67 (1.09-2.57) | 0.03 | 1.69 (1.06-2.71) |
| rs1363448 | *PCDHGA12* | 140783596 | 0.02 | 1.42 (1.06-1.89) | 0.10 | 1.55 (0.91-2.63) |
| rs6861047 | *PCDHGA12* | 140719090 | 0.02 | 0.61 (0.41-0.92) | 0.03 | 0.60 (0.38-0.94) |
| rs998051 | *TCERG1* | 145875259 | 0.02 | 1.77 (1.08-2.88) | 0.03 | 1.77 (1.07-2.95) |
| rs273915 | *SLC22A4* | 131660119 | 0.02 | 0.68 (0.49-0.94) | 0.03 | 0.64 (0.42-0.95) |
| rs173683 | *FCHSD1* | 141028047 | 0.02 | 1.4 (1.04-1.87) | 0.03 | 1.69 (1.04-2.74) |
| rs1421898 | *intergenic* | 158111073 | 0.02 | 1.84 (1.08-3.14) | 0.03 | 1.90 (1.05-3.42) |
| rs4705986 | *ZCCHC10* | 132349654 | 0.03 | 2.11 (1.09-4.07) | 0.03 | 2.11 (1.09-4.07) |
| rs6871729 | *RP11-166A12.1* | 122027048 | 0.03 | 0.46 (0.23-0.90) | 0.03 | 0.46 (0.23-0.92) |
| rs2069885 | *AC011427.1* | 135228165 | 0.03 | 1.53 (1.05-2.24) | 0.02 | 1.67 (1.08-2.59) |
| rs547356 | *intergenic* | 126831288 | 0.03 | 1.50 (1.05-2.14) | 0.02 | 1.62 (1.07-2.46) |
| rs1952657 | *intergenic* | 157777400 | 0.03 | 0.60 (0.39-0.94) | 0.03 | 0.60 (0.37-0.96) |
| rs3749780 | *SLC25A2* | 140682757 | 0.03 | 0.63 (0.43-0.94) | 0.04 | 0.63 (0.40-0.97) |
| rs6892346 | *intergenic* | 154007669 | 0.03 | 1.45 (1.04-2.02) | 0.10 | 1.43 (0.93-2.20) |
| rs274546 | *AC034220.3* | 131699867 | 0.03 | 0.71 (0.53-0.96) | 0.13 | 0.72 (0.48-1.09) |
| RS164080 | *GNPDA1* | 141391532 | 0.03 | 1.37 (1.03-1.81) | 0.10 | 1.51 (0.92-2.46) |
| rs1060740 | *CLINT1* | 157220428 | 0.03 | 0.52 (0.29-0.93) | 0.03 | 0.53 (0.29-0.95) |
| rs904612 | *CTB-35F21.1* | 139070952 | 0.03 | 1.40 (1.03-1.89) | 0.04 | 1.51 (1.01-2.26) |
| rs6891142 | *GRIA1* | 153142662 | 0.03 | 1.92 (1.06-3.46) | 0.03 | 1.92 (1.06-3.46) |
| **rs13179436** | ***PRELID2*** | **144883076** | **0.03** | **1.50 (1.04-2.16)** | **0.09** | **1.44 (0.93-2.23)** |
| rs12186424 | *RP11-114H21.2* | 135825511 | 0.03 | 1.45 (1.03-2.03) | 0.07 | 1.46 (0.97-2.20) |
| rs6861199 | *FAM13B* | 137391124 | 0.03 | 0.67 (0.47-0.96) | 0.05 | 0.65 (0.43-0.99) |
| rs272879 | *SLC22A4* | 131670546 | 0.03 | 0.72 (0.54-0.97) | 0.25 | 0.78 (0.51-1.19) |
| rs970629 | *CTC-575N7.1* | 129118828 | 0.03 | 1.65 (1.04-2.62) | 0.06 | 1.64 (0.98-2.74) |
| rs31336 | *FSTL4* | 132787716 | 0.03 | 0.39 (0.17-0.92) | 0.04 | 0.39 (0.16-0.94) |
| rs272893 | *SLC22A4* | 131663062 | 0.03 | 0.71 (0.52-0.97) | 0.12 | 0.70 (0.45-1.09) |
| rs17208397 | *PCDHGA12* | 140798639 | 0.03 | 0.64 (0.43-0.96) | 0.02 | 0.58 (0.37-0.91) |
| rs2337986 | *AC005609.1* | 140160622 | 0.03 | 0.71 (0.52-0.97) | 0.01 | 0.55 (0.35-0.85) |
| rs283424 | *intergenic* | 153939449 | 0.03 | 2.53 (1.07-5.98) | 0.04 | 2.53 (1.04-6.15) |
| rs251021 | *DIAPH1* | 140899268 | 0.03 | 0.55 (0.32-0.95) | 0.05 | 0.57 (0.32-1.00) |
| rs4704977 | *intergenic* | 155560219 | 0.03 | 0.69 (0.49-0.97) | 0.01 | 0.57 (0.38-0.86) |
| rs325227 | *CTB-57H20.1* | 143150874 | 0.03 | 1.37 (1.02-1.84) | 0.25 | 1.28 (0.83-1.97) |
| rs7722600 | *RP11-381K20.2* | 137194762 | 0.03 | 1.47 (1.02-2.12) | 0.13 | 1.38 (0.91-2.09) |
| rs10069634 | *PRR16* | 119885247 | 0.03 | 1.38 (1.02-1.87) | 0.08 | 1.43 (0.96-2.13) |
| rs2963163 | *intergenic* | 161702081 | 0.03 | 1.65 (1.03-2.64) | 0.07 | 1.58 (0.96-2.62) |
| rs10040489 | *FSTL4* | 132837218 | 0.03 | 1.47 (1.02-2.10) | 0.20 | 1.32 (0.86-2.03) |
| **rs17448496** | ***PPP2R2B*** | **146015615** | **0.04** | **1.47 (1.02-2.12)** | **0.02** | **1.67 (1.09-2.56)** |
| rs10044964 | *RP11-510I6.2* | 120952456 | 0.04 | 0.68 (0.47-0.97) | 0.02 | 0.60 (0.38-0.93) |
| rs700713 | *FSTL4* | 132723619 | 0.04 | 1.55 (1.02-2.37) | 0.05 | 1.57 (0.99-2.49) |
| rs17054760 | *intergenic* | 157036246 | 0.04 | 1.48 (1.02-2.15) | 0.09 | 1.44 (0.93-2.23) |
| rs2304029 | *FAT2* | 150891772 | 0.04 | 0.3 (0.12-0.69) | 0.04 | 0.3 (0.12-0.69) |
| rs4958881 | *TNIP1* | 150450236 | 0.04 | 0.62 (0.40-0.97) | 0.03 | 0.57 (0.34-0.95) |
| rs256775 | *AC011347.1* | 155511486 | 0.04 | 0.72 (0.53-0.98) | 0.04 | 0.64 (0.42-0.97) |
| rs6580194 | *FCHSD1* | 141034123 | 0.04 | 1.35 (1.01-1.82) | 0.04 | 1.55 (1.01-2.39) |
| rs11956128 | *intergenic* | 161464255 | 0.04 | 1.43 (1.01-2.03) | 0.07 | 1.45 (0.96-2.19) |
| rs165355 | *SLC36A1* | 150858126 | 0.04 | 1.42 (1.01-2.01) | 0.02 | 1.65 (1.09-2.49) |
| **rs13170526** | ***EBF1*** | **158175669** | **0.04** | **1.65 (1.01-2.67)** | **0.15** | **1.46 (0.87-2.47)** |
| rs211029 | *GABRG2* | 161533066 | 0.04 | 0.71 (0.51-0.98) | 0.09 | 0.68 (0.44-1.05) |
| rs165330 | *SLC36A1* | 150875468 | 0.04 | 1.39 (1.01-1.91) | 0.01 | 1.71 (1.13-2.58) |
| rs1433048 | *IL12B* | 158755845 | 0.04 | 1.40 (1.01-1.95) | 0.05 | 1.51 (1.00-2.28) |
| rs210989 | *GABRG2* | 161533712 | 0.04 | 0.63 (0.41-0.98) | 0.11 | 0.68 (0.42-1.08) |
| rs4912872 | *AC005592.2* | 142030968 | 0.04 | 0.58 (0.34-0.98) | 0.03 | 0.54 (0.31-0.93) |
| rs17716322 | *CTC-349C3.1* | 134477118 | 0.04 | 0.60 (0.36-0.98) | 0.08 | 0.62 (0.37-1.06) |
| rs31872 | *PCDHA6* | 140372222 | 0.05 | 0.72 (0.52-0.99) | 0.13 | 0.72 (0.48-1.09) |
| rs1431945 | *intergenic* | 121104400 | 0.05 | 1.71 (1.01-2.89) | 0.01 | 2.11 (1.16-3.82) |
| rs12523051 | *GRIA1* | 153087153 | 0.05 | 1.39 (1.00-1.94) | 0.15 | 1.37 (0.89-2.09) |
| rs7701427 | *CAMK2A* | 149632955 | 0.05 | 0.66 (0.44-0.99) | 0.03 | 0.60 (0.37-0.94) |
| rs6893204 | *intergenic* | 156874818 | 0.05 | 1.32 (1.00-1.75) | 0.29 | 1.26 (0.82-1.93) |
| rs6889792 | *RP11-510I6.2* | 120951488 | 0.05 | 0.73 (0.54-0.99) | 0.11 | 0.69 (0.44-1.08) |
| rs255478 | *intergenic* | 151425896 | 0.05 | 0.73 (0.54-1.00) | 0.04 | 0.65 (0.43-0.98) |
| rs11954297 | *RBM27* | 145668298 | 0.05 | 1.38 (0.99-1.92) | 0.03 | 1.57 (1.04-2.35) |
| **rs403151** | ***PPP2R2B*** | **146033408** | **0.06** | **0.70 (0.49-1.00)** | **0.02** | **0.57 (0.37-0.90)** |
| rs7712156 | *intergenic* | 133140038 | 0.06 | 0.60 (0.36-1.01) | 0.05 | 0.54 (0.30-0.98) |
| rs17096590 | *RBM27* | 145693202 | 0.06 | 1.32 (0.98-1.78) | 0.04 | 1.54 (1.02-2.31) |
| rs1551937 | *SNX24* | 122328421 | 0.06 | 1.34 (0.98-1.82) | 0.02 | 1.64 (1.06-2.52) |
| rs17114459 | *intergenic* | 152732763 | 0.06 | 1.47 (0.98-2.19) | 0.03 | 1.67 (1.04-2.67) |
| rs4704727 | *TIMD4* | 156380067 | 0.06 | 1.33 (0.98-1.82) | 0.03 | 1.60 (1.03-2.47) |
| rs4835768 | *intergenic* | 137441767 | 0.06 | 1.31 (0.98-1.75) | 0.04 | 1.65 (1.02-2.67) |
| rs726684 | *PCDHGA12* | 140772427 | 0.06 | 0.69 (0.47-1.02) | 0.05 | 0.64 (0.41-0.99) |
| rs13183976 | *RBM27* | 145594215 | 0.06 | 1.31 (0.98-1.76) | 0.04 | 1.51 (1.01-2.27) |
| rs9327410 | *GRAMD3* | 125826789 | 0.07 | 1.38 (0.98-1.95) | 0.02 | 1.65 (1.07-2.52) |
| rs6860507 | *NIPAL4* | 156898690 | 0.07 | 0.76 (0.58-1.01) | 0.03 | 0.61 (0.39-0.94) |
| rs7729098 | *RBM27* | 145643048 | 0.07 | 1.30 (0.97-1.74) | 0.05 | 1.51 (1.00-2.26) |
| rs10478885 | *ADAMTS19* | 128865864 | 0.08 | 1.42 (0.96-2.12) | 0.05 | 1.59 (1.00-2.53) |
| rs7710890 | *intergenic* | 135009714 | 0.08 | 1.38 (0.96-1.99) | 0.04 | 1.56 (1.01-2.40) |
| rs6868496 | *intergenic* | 150151271 | 0.08 | 1.29 (0.96-1.74) | 0.05 | 1.59 (1.01-2.51) |
| rs3805653 | *FBN2* | 127761510 | 0.08 | 0.71 (0.49-1.04) | 0.04 | 0.63 (0.41-0.98) |
| rs6891051 | *RP11-166A12.1* | 122027308 | 0.09 | 1.30 (0.96-1.76) | 0.04 | 1.56 (1.01-2.40) |
| rs274554 | *SLC22A5* | 131724950 | 0.09 | 1.38 (0.94-2.01) | 0.02 | 1.65 (1.08-2.53) |
| rs12514133 | *KDM3B* | 137742465 | 0.09 | 1.34 (0.95-1.88) | 0.04 | 1.54 (1.01-2.34) |
| rs11738832 | *NRG2* | 139294677 | 0.10 | 1.28 (0.95-1.71) | 0.04 | 1.66 (1.03-2.67) |
| rs11334 | *KDM3B* | 137772296 | 0.10 | 1.33 (0.94-1.86) | 0.05 | 1.52 (1.00-2.30) |
| rs10988 | *RP11-118M9.3* | 145499996 | 0.10 | 1.37 (0.99-1.91) | 0.04 | 1.72 (1.12-2.64) |
| rs2563283 | *RP11-515C16.8* | 140105978 | 0.11 | 0.78 (0.57-1.05) | 0.02 | 0.61 (0.40-0.93) |
| rs809635 | *HARS2* | 140081791 | 0.11 | 0.78 (0.58-1.05) | 0.03 | 0.62 (0.41-0.95) |
| rs2081967 | *intergenic* | 154547570 | 0.12 | 0.77 (0.56-1.06) | 0.04 | 0.65 (0.43-0.97) |
| rs11958868 | *AC005609.2* | 140201420 | 0.13 | 0.78 (0.58-1.07) | 0.04 | 0.63 (0.41-0.96) |
| rs3822346 | *AC005609.2* | 140187322 | 0.13 | 0.78 (0.57-1.07) | 0.02 | 0.60 (0.39-0.92) |
| rs42265 | *intergenic* | 128404226 | 0.16 | 0.81 (0.60-1.08) | 0.04 | 0.62 (0.40-0.97) |
| rs245311 | *CTC-228N24.1* | 127166599 | 0.18 | 1.23 (0.90-1.67) | 0.04 | 1.52 (1.01-2.26) |
| **rs1368298** | ***EBF1*** | **158204425** | **0.26** | **1.22 (0.91-1.64)** | **0.02** | **1.91 (1.12-3.25)** |
| rs707176 | *GRIA1* | 153029960 | 0.32 | 0.86 (0.63-1.16) | 0.05 | 0.66 (0.44-0.99) |
| rs13173233 | *intergenic* | 161912623 | 0.32 | 0.85 (0.62-1.17) | 0.05 | 0.64 (0.42-0.99) |
| **rs1919515** | ***SPOCK1*** | **136822835** | **0.40** | **1.13 (0.84-1.51)** | **0.05** | **1.55 (1.00-2.40)** |
