# Supplementary material for: Fine mapping of a linkage peak with integration of lipid traits identifies novel coronary artery disease genes on chromosome 5
Source: BMC Genet. 2012 Feb 27;13:12. doi: 10.1186/1471-2156-13-12 (PMC3309961; doi:10.1186/1471-2156-13-12)
Supplement: Additional file 8 — Association of SNPs with Degree of Atherosclerosis in the Aorta Samples. Displayed are results of association of SNPs with degree of atherosclerosis in the aorta samples, with each significant SNP listed followed by their genic position, base pair position, p-value, odds ratio, and 95% confidence interval for the genotypic and allelic models. [file 1471-2156-13-12-S8.DOCX]

**Additional File 8**

**Table S1. Association of SNPs with Degree of Atherosclerosis in the Aorta Samples.** Displayed are results of association of SNPs with degree of atherosclerosis in the aorta samples, with each significant SNP listed followed by their genic position, base pair position, p-value, odds ratio, and 95% confidence interval for the genotypic and allelic models.

|  |  |  | **Genotypic** | | **Allelic** | |
| --- | --- | --- | --- | --- | --- | --- |
| **SNP** | **GENE** | **Physical Location** | **P-value** | **OR(CI)** | **P-value** | **OR(CI)** |
| rs1990889 | *ATP10B* | 160097162 | 0.001 | 1.76 (0.79-3.93) | 0.002 | 4.63 (4.63-16.0) |
| rs12516222 | *intergenic* | 136138605 | 0.002 | 3.03 (0.84-10.8) | 0.004 | 4.77 (4.77-20.4) |
| rs2307108 | *FBN2* | 127610002 | 0.002 | 1.87 (0.88-3.95) | 0.01 | 3.39 (3.39-10.2) |
| rs3756366 | *H2AFY* | 134696768 | 0.004 | 0.24 (0.08-0.72) | 0.004 | 0.23 (0.23-0.77) |
| rs250201 | *AC005592.2* | 142033308 | 0.004 | 0.38 (0.15-0.95) | 0.004 | 0.22 (0.22-0.78) |
| rs32946 | *intergenic* | 141129204 | 0.004 | 0.38 (0.15-0.93) | 0.003 | 0.33 (0.33-1.02) |
| rs9327409 | *GRAMD3* | 125826424 | 0.01 | 2.49 (1.05-5.87) | 0.01 | 2.08 (2.08-6.64) |
| rs4912870 | *AC005592.2* | 142015715 | 0.01 | 0.43 (0.19-0.97) | 0.005 | 0.26 (0.26-0.86) |
| rs11242001 | *ADAMTS19* | 128802515 | 0.01 | 0.11 (0.02-0.52) | 0.005 | 0.10 (0.10-0.50) |
| rs2157916 | *intergenic* | 136114752 | 0.01 | 4.66 (1.35-16.0) | 0.01 | 4.61 (4.61-18.0) |
| rs2190578 | *CTB-1I21.2* | 136190484 | 0.01 | 0.06 (0.00-0.66) | 0.05 | 0.06 (0.06-0.66) |
| rs274554 | *SLC22A5* | 131724950 | 0.01 | 0.20 (0.07-0.56) | 0.04 | 0.19 (0.19-0.58) |
| rs28076 | *intergenic* | 136045459 | 0.01 | 2.92 (1.19-7.14) | 0.01 | 5.66 (5.66-21.4) |
| rs456290 | *SGCD* | 156155155 | 0.01 | 0.38 (0.16-0.88) | 0.01 | 0.26 (0.26-0.84) |
| rs1800449 | *SRFBP1* | 121413208 | 0.01 | 0.43 (0.17-1.03) | 0.01 | 0.18 (0.18-0.61) |
| **rs2043478** | ***SPOCK1*** | **136498493** | **0.01** | **0.37 (0.13-1.07)** | **0.01** | **0.29 (0.29-0.97)** |
| rs34012 | *AC005592.2* | 141995777 | 0.01 | 0.17 (0.04-0.74) | 0.03 | 0.17 (0.17-0.72) |
| rs13164781 | *FBN2* | 127870625 | 0.01 | 0.40 (0.16-0.96) | 0.02 | 0.28 (0.28-0.96) |
| rs4836459 | *ADAMTS19* | 128823842 | 0.01 | 0.36 (0.13-0.93) | 0.01 | 0.26 (0.26-0.84) |
| rs7730134 | *intergenic* | 123261310 | 0.01 | 4.02 (1.45-11.1) | 0.01 | 4.45 (4.45-14.4) |
| rs10052876 | *CCNJL* | 159713095 | 0.01 | 3.80 (1.35-10.6) | 0.05 | 5.29 (5.29-18.2) |
| rs28063 | *intergenic* | 136064362 | 0.01 | 1.68 (0.80-3.54) | 0.01 | 3.72 (3.72-11.5) |
| rs7724326 | *ADAMTS19* | 128847172 | 0.01 | 3.10 (1.06-9.03) | 0.01 | 3.59 (3.59-11.2) |
| rs31326 | *FSTL4* | 132818098 | 0.01 | 0.16 (0.03-0.92) | 0.05 | 0.16 (0.16-0.90) |
| rs17164935 | *MEGF10* | 126791282 | 0.01 | 1.72 (0.80-3.71) | 0.03 | 3.31 (3.31-10.2) |
| rs331093 | *FBN2* | 127714019 | 0.01 | 0.23 (0.06-0.85) | 0.07 | 0.22 (0.22-0.85) |
| rs17099156 | *AC005592.2* | 142019596 | 0.01 | 2.29 (1.02-5.10) | 0.01 | 1.90 (1.90-5.66) |
| rs2418543 | *intergenic* | 129628998 | 0.01 | 0.02 (0.00-0.31) | 0.03 | 0.02 (0.02-0.31) |
| rs32927 | *intergenic* | 141122067 | 0.01 | 3.78 (1.35-10.60) | 0.02 | 4.15 (4.15-14.7) |
| rs10071253 | *ADAMTS19* | 128844403 | 0.01 | 3.66 (1.45-9.19) | 0.01 | 5.40 (5.40-17.5) |
| rs190450 | *FBN2* | 127614472 | 0.01 | 0.37 (0.16-0.83) | 0.06 | 0.24 (0.24-0.77) |
| rs32222 | *FBN2* | 127672312 | 0.01 | 0.39 (0.18-0.85) | 0.01 | 0.29 (0.29-0.91) |
| rs10041179 | *GRIA1* | 152916115 | 0.01 | 0.42 (0.19-0.92) | 0.03 | 0.40 (0.40-1.17) |
| rs4476720 | *GRAMD3* | 125799833 | 0.02 | 0.38 (0.12-1.17) | 0.02 | 0.27 (0.27-0.92) |
| **rs1434660** | ***SPOCK1*** | **136484839** | **0.02** | **0.22 (0.07-0.68)** | **0.15** | **0.24 (0.24-0.82)** |
| rs1895172 | *ADAMTS19* | 128874733 | 0.02 | 2.55 (1.14-5.70) | 0.01 | 2.87 (2.87-8.57) |
| rs2190573 | *intergenic* | 136170977 | 0.02 | 0.20 (0.04-0.98) | 0.03 | 0.20 (0.20-0.97) |
| rs248589 | *intergenic* | 141084320 | 0.02 | 0.39 (0.16-0.96) | 0.02 | 0.36 (0.36-1.31) |
| rs6580248 | *AC005592.2* | 141857947 | 0.02 | 0.60 (0.25-1.46) | 0.19 | 0.30 (0.30-0.95) |
| rs1366352 | *intergenic* | 123531570 | 0.02 | 0.42 (0.14-1.22) | 0.01 | 0.23 (0.23-0.92) |
| rs3892475 | *CTD-2131I18.1* | 133249805 | 0.02 | 2.55 (1.08-6.01) | 0.09 | 2.41 (2.41-7.37) |
| rs6596102 | *HSPA4* | 132415569 | 0.02 | 0.19 (0.04-0.87) | 0.03 | 0.18 (0.18-0.83) |
| rs2560040 | *C5orf4* | 154209207 | 0.02 | 0.37 (0.15-0.89) | 0.02 | 0.37 (0.37-1.10) |
| **rs1383167** | ***PPP2R2B*** | **146398383** | **0.02** | **0.17 (0.03-0.87)** | **0.1** | **0.17 (0.17-0.87)** |
| rs6891142 | *GRIA1* | 153142662 | 0.02 | 2.19 (0.82-5.81) | 0.02 | 4.13 (4.13-14.2) |
| **rs6865969** | ***EBF1*** | **158502728** | **0.02** | **4.40 (1.08-17.8)** | **0.35** | **4.40 (4.40-17.8)** |
| rs153477 | *GM2A* | 150639409 | 0.02 | 0.38 (0.16-0.90) | 0.06 | 0.14 (0.14-0.79) |
| rs6860957 | *GRAMD3* | 125801394 | 0.02 | 0.14 (0.02-0.91) | 0.01 | 0.14 (0.14-0.88) |
| rs6861047 | *PCDHGA12* | 140719090 | 0.02 | 0.51 (0.20-1.29) | 0.02 | 0.28 (0.28-0.92) |
| rs248128 | *intergenic* | 126530591 | 0.02 | 3.09 (1.20-7.95) | 0.17 | 3.74 (3.74-12.4) |
| rs6893408 | *AC005592.2* | 142022777 | 0.02 | 1.65 (0.75-3.65) | 0.01 | 3.42 (3.42-10.7) |
| rs168693 | *intergenic* | 126487784 | 0.02 | 0.28 (0.10-0.74) | 0.12 | 0.32 (0.32-1.09) |
| rs6861657 | *intergenic* | 136254338 | 0.02 | 3.01 (1.08-8.42) | 0.01 | 2.24 (2.24-7.17) |
| rs7720917 | *intergenic* | 153864932 | 0.02 | 4.12 (1.07-15.8) | 0.02 | 4.37 (4.37-17.4) |
| **rs11746562** | ***SPOCK1*** | **136499431** | **0.02** | **3.71 (1.38-9.98)** | **0.04** | **5.00 (5.00-17.2)** |
| rs17164094 | *FBN2* | 127850855 | 0.03 | 3.11 (1.12-8.57) | 0.02 | 3.30 (3.30-11.3) |
| rs12152793 | *intergenic* | 122817100 | 0.03 | 5.95 (1.33-26.6) | 0.02 | 5.95 (5.95-26.6) |
| rs154001 | *FBN2* | 127685135 | 0.03 | 5.37 (1.23-23.4) | 0.04 | 5.77 (5.77-26.0) |
| rs32220 | *FBN2* | 127641916 | 0.03 | 0.27 (0.11-0.68) | 0.03 | 0.21 (0.21-0.66) |
| rs938537 | *intergenic* | 160458036 | 0.03 | 0.34 (0.12-0.94) | 0.09 | 0.29 (0.29-0.96) |
| rs17676694 | *FBN2* | 127680042 | 0.03 | 2.79 (1.01-7.73) | 0.02 | 3.86 (3.86-12.8) |
| rs11242026 | *intergenic* | 129873659 | 0.03 | 3.01 (1.21-7.43) | 0.07 | 4.92 (4.92-16.7) |
| rs247456 | *intergenic* | 133374511 | 0.03 | 2.23 (0.97-5.12) | 0.03 | 3.46 (3.46-11.3) |
| rs6868664 | *FBN2* | 127742721 | 0.03 | 2.40 (1.02-5.65) | 0.06 | 3.04 (3.04-10.0) |
| rs3756325 | *PCDHA6* | 140368859 | 0.03 | 2.43 (1.00-5.95) | 0.02 | 2.72 (2.72-8.64) |
| rs1432812 | *5S_rRNA* | 155274700 | 0.03 | 0.19 (0.04-0.90) | 0.01 | 0.19 (0.19-0.89) |
| rs153977 | *FBN2* | 127677581 | 0.03 | 0.31 (0.11-0.87) | 0.03 | 0.31 (0.31-0.95) |
| rs12523624 | *AC005592.2* | 142020508 | 0.03 | 0.58 (0.29-1.17) | 0.1 | 0.24 (0.24-0.75) |
| **rs6596384** | ***SPOCK1*** | **136802547** | **0.03** | **0.21 (0.05-0.80)** | **0.12** | **0.21 (0.21-0.80)** |
| **rs1235399** | ***SPOCK1*** | **136733738** | **0.03** | **3.24 (1.39-7.53)** | **0.09** | **3.38 (3.38-11.7)** |
| rs441396 | *intergenic* | 153312211 | 0.03 | 0.29 (0.10-0.85) | 0.12 | 0.20 (0.20-0.68) |
| **rs161042** | ***PPP2R2B*** | **146185874** | **0.03** | **0.19 (0.04-0.83)** | **0.16** | **0.19 (0.19-0.82)** |
| rs157664 | *SGCD* | 156123995 | 0.03 | 0.32 (0.13-0.79) | 0.03 | 0.33 (0.33-1.04) |
| rs4912691 | *CTC-367F4.1* | 144607666 | 0.03 | 2.14 (1.03-4.46) | 0.03 | 2.03 (2.03-6.40) |
| rs13161129 | *FSTL4* | 132830294 | 0.03 | 0.39 (0.18-0.86) | 0.07 | 0.39 (0.39-1.17) |
| rs17208397 | *PCDHGA12* | 140798639 | 0.03 | 1.69 (0.78-3.64) | 0.03 | 3.22 (3.22-9.99) |
| rs6865020 | *intergenic* | 129820918 | 0.03 | 3.33 (1.11-10.0) | 0.11 | 2.57 (2.57-9.47) |
| **rs17645325** | ***EBF1*** | **158476709** | **0.03** | **3.49 (1.33-9.11)** | **0.03** | **3.16 (3.16-9.99)** |
| rs1036199 | *HAVCR2* | 156531736 | 0.03 | 3.27 (1.17-9.14) | 0.03 | 4.57 (4.57-14.8) |
| **rs4921252** | ***EBF1*** | **158473524** | **0.03** | **0.36 (0.15-0.89)** | **0.03** | **0.38 (0.38-1.17)** |
| rs979923 | *intergenic* | 129603494 | 0.03 | 2.62 (1.06-6.46) | 0.03 | 4.02 (4.02-13.2) |
| rs12652669 | *SGCD* | 155876204 | 0.03 | 0.30 (0.10-0.90) | 0.11 | 0.21 (0.21-0.85) |
| rs9687567 | *GRAMD3* | 125800646 | 0.04 | 3.86 (1.43-10.3) | 0.02 | 4.50 (4.50-14.6) |
| **rs13182446** | ***PPP2R2B*** | **146311790** | **0.04** | **0.21 (0.04-0.98)** | **0.17** | **0.21 (0.21-0.98)** |
| rs246962 | *intergenic* | 118915629 | 0.04 | 2.90 (1.08-7.79) | 0.05 | 3.33 (3.33-9.89) |
| rs791326 | *intergenic* | 126958230 | 0.04 | 0.40 (0.19-0.85) | 0.03 | 0.43 (0.43-1.33) |
| rs11955126 | *STK32A* | 146720355 | 0.04 | 0.70 (0.32-1.49) | 0.02 | 0.28 (0.28-0.86) |
| **rs7732511** | ***EBF1*** | **158530015** | **0.04** | **4.10 (1.49-11.2)** | **0.04** | **3.32 (3.32-10.1)** |
| rs1978235 | *intergenic* | 129921388 | 0.04 | 2.70 (1.15-6.34) | 0.14 | 6.25 (6.25-23.6) |
| **rs6863244** | ***SPOCK1*** | **136335948** | **0.04** | **3.39 (1.20-9.56)** | **0.21** | **2.57 (2.57-8.05)** |
| rs284439 | *SGCD* | 156172418 | 0.04 | 2.18 (0.96-4.95) | 0.03 | 6.37 (6.37-25.1) |
| **rs718703** | ***SPOCK1*** | **136403289** | **0.04** | **0.16 (0.03-0.93)** | **0.04** | **0.16 (0.16-0.91)** |
| **rs17464441** | ***PPP2R2B*** | **146438829** | **0.04** | **2.39 (0.97-5.85)** | **0.04** | **8.09 (8.09-39.8)** |
| rs10463889 | *intergenic* | 131361916 | 0.04 | 1.85 (0.86-3.95) | 0.02 | 4.12 (4.12-14.0) |
| rs1049171 | *STK32A* | 146771978 | 0.04 | 0.67 (0.32-1.40) | 0.05 | 0.23 (0.23-0.79) |
| **rs7736604** | ***PPP2R2B*** | **146068662** | **0.04** | **0.28 (0.09-0.85)** | **0.02** | **0.28 (0.28-0.94)** |
| rs157670 | *SGCD* | 156113221 | 0.04 | 0.23 (0.09-0.59) | 0.04 | 0.20 (0.20-0.68) |
| rs17626237 | *FSTL4* | 132625143 | 0.04 | 2.65 (1.04-6.72) | 0.08 | 2.92 (2.92-9.76) |
| rs12163993 | *PHF15* | 133887780 | 0.04 | 4.67 (1.44-15.1) | 0.04 | 3.39 (3.39-11.5) |
| rs3805635 | *FBN2* | 127673121 | 0.04 | 0.2 (0.06-0.59) | 0.05 | 0.16 (0.16-0.53) |
| rs906678 | *intergenic* | 150779040 | 0.04 | 0.34 (0.15-0.75) | 0.23 | 0.24 (0.24-0.73) |
| rs17167632 | *intergenic* | 133930528 | 0.04 | 1.72 (0.80-3.70) | 0.04 | 3.31 (3.31-10.2) |
| rs187269 | *GABRB2* | 160756625 | 0.04 | 0.51 (0.20-1.30) | 0.14 | 0.24 (0.24-0.81) |
| rs11948613 | *SAR1B* | 133947011 | 0.04 | 3.65 (1.35-9.88) | 0.04 | 4.76 (4.76-16.2) |
| rs6887366 | *HTR4* | 147851270 | 0.04 | 1.67 (0.71-3.92) | 0.12 | 3.65 (3.65-11.9) |
| **rs4976408** | ***SPOCK1*** | **136431804** | **0.04** | **1.94 (0.86-4.40)** | **0.09** | **3.23 (3.23-10.3)** |
| **rs6872714** | ***SPOCK1*** | **136319856** | **0.04** | **0.38 (0.12-1.13)** | **0.16** | **0.27 (0.27-0.93)** |
| rs340051 | *GRXCR2* | 145235121 | 0.04 | 0.38 (0.17-0.88) | 0.12 | 0.47 (0.47-1.38) |
| rs4835728 | *DNAJC18* | 138754741 | 0.04 | 0.50 (0.20-1.21) | 0.04 | 0.30 (0.30-0.97) |
| rs552850 | *FBN2* | 127700694 | 0.04 | 0.24 (0.08-0.72) | 0.25 | 0.26 (0.26-0.89) |
| rs4958553 | *intergenic* | 151903171 | 0.04 | 0.14 (0.02-0.86) | 0.13 | 0.14 (0.14-0.86) |
| rs9313774 | *intergenic* | 157827241 | 0.04 | 2.93 (1.10-7.80) | 0.03 | 2.45 (2.45-7.56) |
| rs2041318 | *intergenic* | 126438360 | 0.04 | 3.71 (1.47-9.32) | 0.37 | 4.58 (4.58-15.5) |
| **rs17600115** | ***SPOCK1*** | **136420952** | **0.04** | **0.22 (0.05-0.88)** | **0.09** | **0.23 (0.23-1.11)** |
| **rs249907** | ***PPP2R2B*** | **146150857** | **0.04** | **0.38 (0.11-1.26)** | **0.07** | **0.25 (0.25-0.94)** |
| rs3749780 | *SLC25A2* | 140682757 | 0.04 | 2.63 (1.26-5.50) | 0.01 | 4.90 (4.90-17.0) |
| rs17060660 | *intergenic* | 162108705 | 0.05 | 3.75 (1.06-13.2) | 0.06 | 3.83 (3.83-14.3) |
| rs700713 | *FSTL4* | 132723619 | 0.05 | 2.17 (0.90-5.21) | 0.03 | 3.29 (3.29-10.8) |
| rs10155614 | *GRIA1* | 153148731 | 0.05 | 0.36 (0.15-0.84) | 0.05 | 0.45 (0.45-1.47) |
| **rs13170526** | ***EBF1*** | **158175669** | **0.05** | **2.02 (0.84-4.85)** | **0.05** | **3.44 (3.44-10.7)** |
| rs31902 | *intergenic* | 144352050 | 0.05 | 2.01 (0.87-4.67) | 0.09 | 3.51 (3.51-11.3) |
| rs10793817 | *FSTL4* | 132838301 | 0.06 | 2.38 (0.96-5.92) | 0.01 | 4.30 (4.30-13.9) |
| rs11167643 | *intergenic* | 153222799 | 0.06 | 1.90 (0.88-4.11) | 0.04 | 4.14 (4.14-13.1) |
| rs6596097 | *HSPA4* | 132392393 | 0.06 | 0.30 (0.10-0.88) | 0.04 | 0.23 (0.23-0.78) |
| rs6556349 | *intergenic* | 157879264 | 0.06 | 2.39 (0.85-6.69) | 0.01 | 3.52 (3.52-11.8) |
| rs35524 | *MEGF10* | 126701622 | 0.06 | 1.90 (0.94-3.85) | 0.04 | 3.79 (3.79-12.6) |
| rs10069900 | *GABRB2* | 160917953 | 0.06 | 2.92 (1.31-6.53) | 0.01 | 3.83 (3.83-12.6) |
| rs253700 | *AC008565.1* | 120374037 | 0.06 | 2.11 (0.98-4.56) | 0.02 | 3.44 (3.44-10.9) |
| rs1644305 | *intergenic* | 133203596 | 0.06 | 2.8 (1.23-6.36) | 0.01 | 6.70 (6.70-30.7) |
| rs9327410 | *GRAMD3* | 125826789 | 0.07 | 0.42 (0.18-0.97) | 0.05 | 0.44 (0.44-1.37) |
| rs264122 | *intergenic* | 129647781 | 0.07 | 0.15 (0.03-0.79) | 0.03 | 0.14 (0.14-0.75) |
| rs12110208 | *intergenic* | 151601252 | 0.08 | 5.64 (2.00-15.9) | 0.03 | 7.25 (7.25-25.0) |
| **rs443033** | ***PRELID2*** | **145210041** | **0.08** | **3.15 (1.09-9.12)** | **0.04** | **2.70 (2.70-9.19)** |
| rs6872196 | *RP11-541P9.3* | 162582264 | 0.08 | 0.36 (0.14-0.94) | 0.05 | 0.26 (0.26-0.81) |
| rs3210047 | *CTC-203F4.1* | 134670765 | 0.09 | 0.42 (0.19-0.94) | 0.04 | 0.46 (0.46-1.30) |
| rs7707445 | *intergenic* | 156449748 | 0.09 | 4.10 (1.10-15.2) | 0.04 | 3.34 (3.34-14.9) |
| rs3910240 | *NRG2* | 139320005 | 0.09 | 2.19 (1.02-4.71) | 0.04 | 2.54 (2.54-8.58) |
| RS413185 | *ACSL6* | 131315225 | 0.1 | 0.47 (0.22-1.02) | 0.02 | 0.20 (0.20-0.71) |
| rs778831 | *GRIA1* | 152996991 | 0.1 | 3.35 (1.11-10.0) | 0.03 | 2.93 (2.93-10.1) |
| rs1859067 | *intergenic* | 134998909 | 0.1 | 0.52 (0.21-1.29) | 0.04 | 0.29 (0.29-0.92) |
| rs4515290 | *JAKMIP2* | 147118578 | 0.11 | 0.40 (0.12-1.29) | 0.05 | 0.27 (0.27-0.99) |
| rs975385 | *AC005592.2* | 141858677 | 0.11 | 0.37 (0.14-0.94) | 0.02 | 0.33 (0.33-0.99) |
| rs299208 | *DTWD2* | 118325429 | 0.11 | 0.28 (0.08-0.98) | 0.03 | 0.27 (0.27-1.04) |
| **rs4704952** | ***EBF1*** | **158127115** | **0.11** | **0.14 (0.03-0.61)** | **0.04** | **0.12 (0.12-0.58)** |
| rs921782 | *PRR16* | 119835711 | 0.12 | 2.58 (1.03-6.47) | 0.02 | 2.68 (2.68-8.39) |
| rs10039306 | *intergenic* | 160306956 | 0.12 | 3.15 (1.12-8.84) | 0.04 | 4.49 (4.49-14.7) |
| rs7732903 | *intergenic* | 136227992 | 0.12 | 0.28 (0.08-0.97) | 0.04 | 0.19 (0.19-0.69) |
| rs7715848 | *intergenic* | 156401478 | 0.13 | 4.47 (1.13-17.6) | 0.05 | 5.05 (5.05-21.8) |
| rs10063647 | *CTC-228N24.3* | 127371588 | 0.13 | 0.02 (0.00-0.30) | 0.01 | 0.02 (0.02-0.30) |
| rs184949 | *RAPGEF6* | 130815593 | 0.14 | 0.40 (0.17-0.96) | 0.04 | 0.44 (0.44-1.31) |
| **rs11167951** | ***PPP2R2B*** | **146363345** | **0.16** | **4.25 (1.35-13.3)** | **0.04** | **4.68 (4.68-16.0)** |
| rs2910291 | *GABRB2* | 160812482 | 0.16 | 0.38 (0.16-0.88) | 0.02 | 0.42 (0.42-1.24) |
| rs26006 | *FNIP1* | 130982768 | 0.16 | 0.39 (0.14-1.11) | 0.04 | 0.26 (0.26-0.88) |
| rs2066949 | *GABRB2* | 160957960 | 0.16 | 0.37 (0.14-0.93) | 0.04 | 0.25 (0.25-0.81) |
| rs6897488 | *GRAMD3* | 125769679 | 0.16 | 1.80 (0.81-4.00) | 0.02 | 3.33 (3.33-10.4) |
| rs32101 | *FNIP1* | 131033900 | 0.16 | 0.43 (0.19-0.97) | 0.04 | 0.60 (0.60-1.81) |
| rs13359174 | *intergenic* | 135942635 | 0.16 | 0.17 (0.03-0.87) | 0.02 | 0.17 (0.17-0.87) |
| rs3756287 | *FNIP1* | 131135478 | 0.18 | 2.74 (1.31-5.74) | 0.04 | 5.92 (5.92-20.9) |
| rs12189066 | *CHSY3* | 129293427 | 0.21 | 0.58 (0.25-1.34) | 0.04 | 0.26 (0.26-0.91) |
| rs1422870 | *RAPGEF6* | 130942624 | 0.21 | 2.39 (1.13-5.03) | 0.03 | 1.77 (1.77-5.92) |
| rs578637 | *AC034228.7* | 131267609 | 0.22 | 2.43 (0.96-6.14) | 0.05 | 3.74 (3.74-12.8) |
| rs10036623 | *GRIA1* | 152919943 | 0.22 | 0.48 (0.14-1.56) | 0.03 | 0.18 (0.18-0.83) |
| **rs867715** | ***PRELID2*** | **144944249** | **0.24** | **0.28 (0.08-0.97)** | **0.03** | **0.31 (0.31-1.21)** |
| rs155820 | *PCDHA6* | 140307746 | 0.27 | 3.85 (1.16-12.7) | 0.04 | 4.21 (4.21-14.7) |
| rs2337986 | *AC005609.1* | 140160622 | 0.3 | 0.34 (0.12-0.96) | **0.02** | 0.39 (0.39-1.27) |
| rs11167489 | *ANKHD1* | 139891089 | 0.36 | 1.63 (0.74-3.57) | 0.03 | 3.16 (3.16-9.83) |
