# Supplementary figures and images for: Fine mapping of a linkage peak with integration of lipid traits identifies novel coronary artery disease genes on chromosome 5
Source: BMC Genet. 2012 Feb 27;13:12. doi: 10.1186/1471-2156-13-12 (PMC3309961; doi:10.1186/1471-2156-13-12)

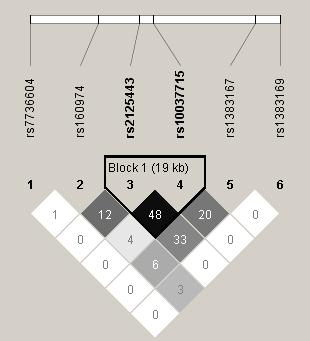

Supplement: Additional file 9 — Linkage disequilibrium patterns of select SNPs in PPP2R2B. LD pattern using Haploview, with r-squared values displayed within each box and the shading of the boxes corresponding do D' values. [file 1471-2156-13-12-S9.JPEG]

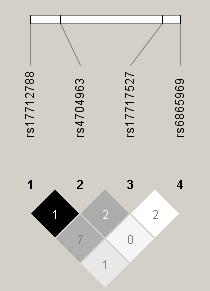

Supplement: Additional file 10 — Linkage disequilibrium patterns of select SNPs in EBF1. LD pattern using Haploview, with r-squared values displayed within each box and the shading of the boxes corresponding do D' values. [file 1471-2156-13-12-S10.JPEG]

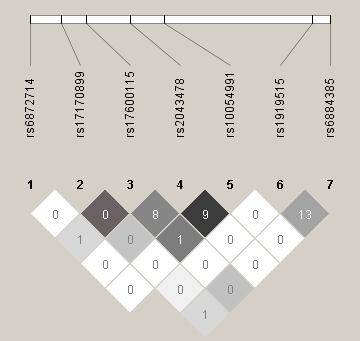

Supplement: Additional file 11 — Linkage disequilibrium patterns of select SNPs in SPOCK1. LD pattern using Haploview, with r-squared values displayed within each box and the shading of the boxes corresponding do D' values. [file 1471-2156-13-12-S11.JPEG]

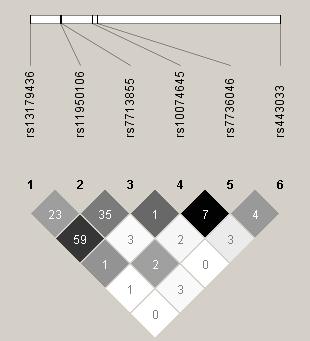

Supplement: Additional file 12 — Linkage disequilibrium patterns of select SNPs in PRELID2. LD pattern using Haploview, with r-squared values displayed within each box and the shading of the boxes corresponding do D' values. [file 1471-2156-13-12-S12.JPEG]
